# Extraction, Purification, Structural Characterization, Biological Activities and Applications in Food of Polysaccharides from *Physalis alkekengi* L. Var. Franchetii (Mast.) Makino

**DOI:** 10.3390/foods15122064

**Published:** 2026-06-07

**Authors:** Han Di, Xinxin Chen, Gang Wang, Ran Chen, Yanhong Wang, Feng Guan

**Affiliations:** 1 School of Pharmacy, Heilongjiang University of Chinese Medicine, Harbin 150040, China; 2 Key Laboratory of Basic and Application Research of Beiyao, Ministry of Education, Heilongjiang University of Chinese Medicine, Harbin 150040, China

**Keywords:** *Physalis alkekengi* L. var. franchetii (Mast.) Makino, polysaccharide, extraction, purification, structural characterization, biological activities

## Abstract

*Physalis alkekengi* L. var. franchetii (Mast.) Makino (*P. alkekengi*) is a well-known traditional Chinese medicine (TCM) with dual edible and medicinal values. Its polysaccharides (PAPs), as core bioactive constituents, have drawn growing research interest amid advances in natural product studies, whereas systematic summaries of existing evidence remain insufficient. This paper collects and analyzes recent progress regarding PAPs extraction, purification, structural characterization, biological activities and targeted applications in diverse food matrices. Current bottlenecks restricting PAPs development are also discussed from multiple research perspectives. Major research outcomes reveal that diverse extraction and purification techniques determine PAPs yield and structural heterogeneity, and characterized structural features are closely associated with their antioxidant, anti-inflammatory, immunomodulatory and other bioactivities. Inadequate mechanistic exploration, incomplete toxicological evaluation and immature industrial extraction systems are critical obstacles limiting further translational application of PAPs. Targeted future research directions are accordingly proposed to address these gaps. This work provides a comprehensive reference and theoretical support for deeper investigation, development and utilization of PAPs as functional food ingredients or herbal bioactive agents.

## 1. Introduction

**Figure 1:**
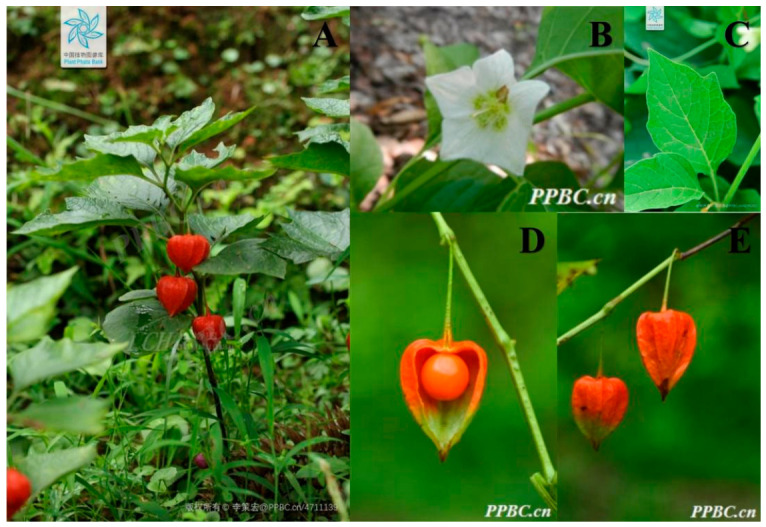
Botanical characteristics of *P. alkekengi*: (**A**) whole plant; (**B**) flowers; (**C**) leaves; (**D**) calyx and fruit; (**E**) fruits. (The plant images in this article are from the Plant Photo Bank of China).

*P. alkekengi*, a perennial herb in the Solanaceae family [1], is used medicinally in the form of its dried calyx or calyx with fruit [2]. *P. alkekengi* is widely distributed in Asia and Europe. In China, it is widely distributed across the northern, northeastern, and northwestern regions, including specific areas such as Shandong Province and Inner Mongolia [3]. Its typical habitats include mountainsides, roadsides, and grasslands. The genus Physalis comprises approximately 120 species worldwide, with 5 species and 2 varieties distributed in China [4]. Among them, *P. alkekengi* has become a research focus due to its significant medicinal value. Its botanical characteristics are shown in Figure 1.

In TCM, it is known under several names, including Suan Jiang, Hong Gu Niang, and Jin Deng Long [5]. *P. alkekengi* is traditionally considered to have a cold property and bitter flavor, with its therapeutic effects primarily targeting the Lung Meridian. Both the whole plant and its fruit are used medicinally. Clinically, it is commonly used for sore throat with hoarseness, cough due to phlegm-heat, difficult urination, and painful dysuria caused by heat [6]. For external use, it is applied in the treatment of pemphigus and eczema. *P. alkekengi* is a plant with a long history of dual use for both medicine and food in China, with its application dating back to around 1800 years ago [7]. Its earliest record appears in the classic Shen Nong Ben Cao Jing, where it is described to have a sour and neutral taste, to treat heat and fullness, to calm the spirit and boost qi, to promote urination, and to facilitate childbirth. It is also known as Cu Jiang. As recorded in the Compendium of Materia Medica, it is bitter and cold in property, non-toxic, and acts to alleviate heat-induced dysphoria and fullness while also calming the mind and tonifying qi [8].

Phytochemical studies have shown that *P. alkekengi* is rich in various bioactive components such as flavonoids [9], terpenoids [10], steroids and polysaccharides [11], and has pharmacological effects such as anti-inflammation [12], antibacterial and immunomodulatory [13]. Among these, PAPs have emerged as a research hotspot in the field of natural products due to their structural diversity, excellent biocompatibility, and low toxicity.

Modern pharmacological studies have confirmed that PAPs exhibit multiple biological activities, such as immunomodulation, blood glucose regulation, and anti-inflammatory and antioxidant effects [14].

*P. alkekengi* not only possesses excellent medicinal value but also demonstrates outstanding edible value and natural nutritional advantages. Its fruit features a unique, pleasantly sweet-and-sour flavor with a well-balanced taste, and is rich in various vitamins, minerals, and natural functional substances [15]. Owing to its distinctive sensory qualities and abundant nutritional components, *P. alkekengi* fruit can be widely applied in fresh consumption, processed snack foods, functional beverages, and baked products. These characteristics align well with the current consumer trend toward natural, healthy, and functional foods, indicating promising prospects for food research and industrial application.

**Figure 2:**
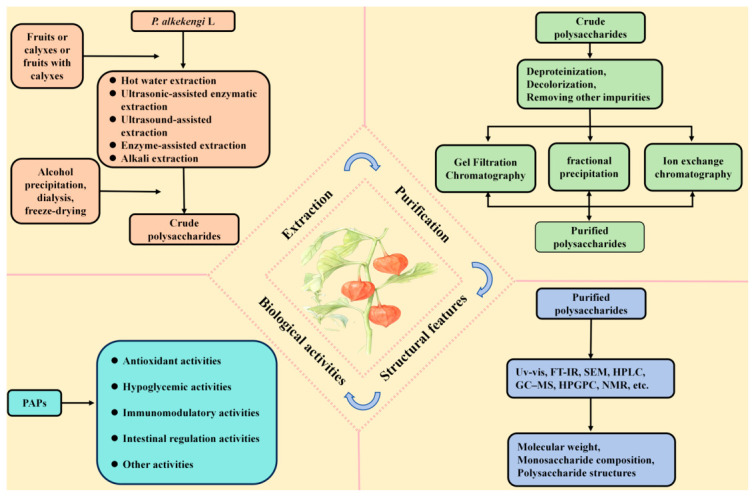
Schematic diagram of extraction, purification, structural features, and biological activities of PAPs.

Although significant progress has been made in the research of PAPs, the existing studies mainly focus on the extraction, purification and various pharmacological activities of PAPs, which contrasts sharply with the lack of basic toxicological data. This review summarizes existing research regarding PAPs-oriented food-grade preparation, stability evaluation and matrix-adapted application cases, clarifying the independent functional role of PAPs in different food systems. It further identifies key limitations, including insufficient industrial-scale trials, unclear stability performance under food processing conditions, and lack of standardized addition criteria for PAP-based food additives. By sorting out these unresolved issues, this work provides theoretical support for developing PAP-based natural preservatives and prebiotic ingredients, guides quality control in food industrial production, and offers a reference for resource-utilization-related policy formulation. Moreover, the structural complexity of PAPs, such as the type of glycosidic bond, branching degree, and the structure-activity relationship between spatial conformation and activity, has not been systematically clarified. Additionally, the standardization of extraction processes and the in-depth analysis of the mechanism of action remain the bottlenecks of current research. This paper presents a comprehensive review of the extraction and purification methods, structural characterization techniques, and biological activity research progress of PAPs worldwide in recent years. Specifically, the extraction methods include hot water extraction (HWE) [16], ultrasonic-assisted extraction (UAE), and enzymatic extraction (EAE) [17]; the purification process employs column chromatography; and the structural characterization methods encompass chromatography, spectroscopy, and nuclear magnetic resonance (NMR) technology [18]. Additionally, it discusses the deficiencies in toxicological research and other related aspects. It aims to provide a theoretical basis and reference for the in-depth development and clinical application of PAPs. A detailed schematic diagram of the extraction method, purification, structural characteristics, and biological activities of PAPs is shown in Figure 2.

**Figure 3:**
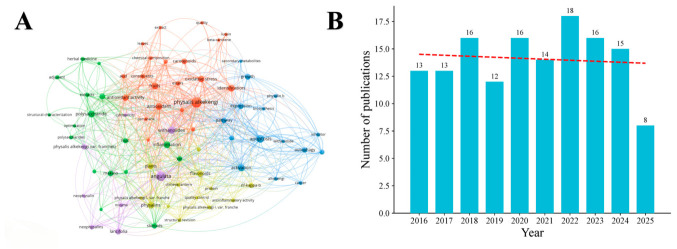
Bibliometric analysis of global research on *P. alkekengi* from 2016 to 2025. (**A**) Keyword co-occurrence network generated by VOSviewer 1.6.20, where node size represents keyword frequency, line thickness indicates co-occurrence intensity, and different colors denote distinct research clusters. (**B**) Annual publications output trend with fitted growth curve based on Web of Science Core Collection data.

A bibliometric analysis of the research on *P. alkekengi* can systematically and objectively reveal its development trajectory, research hotspots, and future trends. By searching Web of Science and performing quantitative and visual analyses of the relevant literature, including publication year, country, institution, authors, and keywords, the annual publication output over the past decade from 2016 to 2025 reveals a relatively stable, fluctuating trend, with the number of publications ranging between 12 and 18 per year and a slight peak in 2022, as shown in Figure 3. This pattern reflects sustained academic interest rather than a dramatic surge in global attention toward this traditional medicinal plant. Analysis of keyword co-occurrence and emergence delineates a clear evolution in research themes. Early studies primarily focused on foundational areas, such as chemical composition, pharmacological activity, including antioxidation and anti-inflammation, and quality control, aiming to clarify the material basis of its traditional medicinal uses. In contrast, recent research frontiers have shifted towards elucidating molecular mechanisms, particularly cellular processes such as apoptosis and autophagy via signaling pathways like NF-κB, while simultaneously exploring potential new applications such as anti-tumor therapy and metabolic disease intervention. Overall, the bibliometric mapping vividly outlines the field’s dynamic progression from basic constituent identification and quality assessment to modern mechanistic exploration and diversified translational applications. This analysis provides future researchers with valuable data support and a macro-level perspective for identifying key scientific questions, seeking collaborative opportunities, and planning innovative research directions. The corresponding bibliometric atlas is shown in Figure 3.

## 2. Extraction Methods of PAPs

At present, there are many methods for extracting polysaccharides from TCM [19]. However, polysaccharides are polar macromolecular substances that are easily soluble in water but poorly soluble in ethanol. Therefore, the most traditional and classic method is the HWE method [20]. Based on the principle of like dissolves like, the polysaccharides are dissolved in water or other polar solvents to complete the extraction operation [21]. In recent years, various novel extraction technologies have emerged. Currently, common techniques for extracting PAPs include HWE, UAE, EAE and ultrasonic-assisted enzymatic extraction (UAEE), with UAEE being the most recommended overall due to its high yield, mild conditions and green characteristics, whereas single-technology approaches have obvious drawbacks and limitations that require targeted optimization strategies.

### 2.1. Hot Water Extraction Method

The HWE method represents the most traditional and commonly employed technique for extracting polysaccharides from TCM [22].

The study investigated three key factors, namely temperature, solid-to-liquid ratio and extraction time, using an orthogonal experimental design. The finding indicates that the optimal extraction conditions consist of a temperature of 90 °C, a duration of 6 h, and a solid-to-liquid ratio of 1:15 (g/mL). Under these optimized parameters, the extraction yield of PAPs reaches its maximum value of 2.920% [17]. Wu et al. processed 3.5 kg of *P. alkekengi* with 2 L of distilled water for 3 h using a previously reported method with modifications. Following the steps, including alcohol precipitation, the yield of PAPs was found to be 10.96% [16]. However, when extracting polysaccharides using hot water, a large amount of other water-soluble impurities will also be dissolved [23]. Subsequent purification steps will be required, and this method is suitable for most neutral polysaccharides and some acidic polysaccharides.

Major limitations of HWE include long extraction time, high energy consumption, low yield and risk of polysaccharide structural degradation under prolonged high-temperature treatment. To improve HWE performance, auxiliary physical fields such as low-power ultrasound or microwave can be combined to shorten extraction time and reduce thermal damage.

### 2.2. Ultrasonic-Assisted Enzymatic Extraction

UAEE is an efficient and environmentally friendly technology for isolating active components from TCM. By combining the cavitation effect of ultrasound with the biocatalytic hydrolysis of enzymes, this method significantly enhances both the extraction efficiency and the quality of the obtained polysaccharides [24].

The researchers employed an ultrasound-assisted cellulase method to optimize the extraction of polysaccharides from the fruit of *P. alkekengi*. Using a combination of a completely randomized design, Plackett-Burman experimental design, and response surface methodology (RSM), the key factors influencing PAPs yield were identified and their optimal levels were determined. The experimental results demonstrate that under the optimized conditions of a 1:15.6 (g/mL) solid-to-liquid ratio, 3.4% cellulase dosage, 30 min of ultrasound at 50 °C, and 60 min of enzymatic hydrolysis at pH 5.0, the average yield of PAPs was 13.78% [25].

The researchers adopted UAEE and optimized the extraction process through RSM. Based on the results of the single-factor experiment, three key factors were selected: the liquid–solid ratio, the concentration of thermostable *α*-amylase, and the concentration of dispase, and the three-factor and three-level experiments were conducted through the Box–Behnken design. The results show that the optimal extraction conditions are when the solid-to-liquid ratio is 1:30 (g/mL), the concentration of thermostable *α*-amylase is 2000 U/mL, and the concentration of dispase is 20,000 U/mL, and the average yield of the obtained product reaches 8.74% [26].

Despite its highest extraction yield, UAEE suffers from high enzyme cost, strict pH and temperature requirements for enzyme activity, and unstable synergistic effects between ultrasound and enzymes. Improvements can be achieved by screening high-efficiency compound enzymes, developing thermostable enzymes, and optimizing ultrasound–enzyme coupling parameters to reduce production cost and enhance process stability.

### 2.3. Ultrasound-Assisted Extraction

UAE is a non-thermal physical extraction technique that employs ultrasonic waves to disrupt cell structures and efficiently extract target polysaccharides from TCM with higher speed and yield [27]. It holds significant application potential in polysaccharide extraction from TCM [20].

Researchers investigated three key factors, namely ultrasonic intensity, extraction duration, and solid-to-liquid ratio (g/mL), by employing an orthogonal experimental design. Results indicated that ultrasonic intensity exerted the most statistically significant effect on the extraction yield of PAPs. The optimal extraction parameters were determined to be an ultrasonic intensity of 300 W, an extraction time of 25 min, and a solid-to-liquid ratio of 1:15 (g/mL). Under these optimized conditions, the extraction yield of PAPs reached 3.67%, which confirms that ultrasonic waves can effectively disrupt cell wall structures and facilitate the release of polysaccharides into the extraction solvent [17].

Limitations of single UAE include limited cell wall destruction capacity and moderate extraction yield. Future improvements can focus on coupling UAE with mild enzymatic treatment or microwave-assisted treatment to strengthen cell wall disruption and further improve extraction efficiency.

### 2.4. Enzyme-Assisted Extraction

The EAE method enhances extraction efficiency by utilizing specific enzymes to catalyze the degradation of plant cell walls. This process reduces extraction resistance and increases the dissolution rate of target compounds. The study has evaluated the extraction effects of neutral protease, papain and alkaline protease. Among them, papain performed most prominently, with its extraction rate far exceeding that of other methods, reaching 8.581% [17].

EAE shows relatively high yield but has long enzymatic hydrolysis time, strict reaction environment requirements, and poor efficiency in breaking dense plant cell walls. It can be improved by combining low-intensity ultrasound pretreatment to destroy cell walls and shorten the enzymatic reaction time.

### 2.5. Alkali Extraction Method

In the alkali extraction method (AEM), polysaccharides are solubilized through the application of a dilute alkaline solution, which hydrolyzes certain glycosidic linkages and disrupts associative interactions with proteins [28]. This facilitates the release of structurally complex polysaccharides, thereby improving extraction efficiency. As an illustration, a reported procedure involving triple extraction of *P. alkekengi* stems with 0.1 mol/L NaOH at 100 °C afforded a polysaccharide yield of 3.4% [18]. This method, however, is subject to significant limitations. A notable drawback is the high impurity content of the crude extract, which increases viscosity and hinders filtration, posing challenges for subsequent processing steps. Furthermore, the alkaline medium can impart a persistent undesirable taste and promote browning, detrimentally affecting the sensory attributes and appearance of the final product.

To mitigate these disadvantages, mild-alkali conditions or short-time alkali treatment are recommended, combined with neutralization and refined purification steps to reduce impurity levels and sensory defects.

Overall, UAEE is identified as the optimal extraction method for PAPs, balancing high extraction yield, mild reaction conditions, green processing characteristics and good retention of polysaccharide bioactivities compared with other approaches.

### 2.6. Comparison of Different Extraction Methods

The aforementioned extraction methods can be classified into three categories: thermal technologies (HWE and AEM), non-thermal technologies (UAE and EAE), and hybrid non-thermal/enzymatic technology (UAEE). Thermal extraction approaches are technically simple and suitable for neutral polysaccharides, but they suffer from high energy consumption, long processing time, co-extraction of non-target impurities, and structural degradation of heat-sensitive PAPs. Non-thermal strategies, such as UAE, feature rapid extraction and low overall operating temperatures, which better preserve the bioactivities of polysaccharides, despite the possibility of local overheating induced by ultrasonic cavitation. EAE is mild and highly selective; however, its practical application is constrained by high enzyme costs and strict sensitivity to pH and temperature. As a representative hybrid technology, UAEE combines ultrasonic cavitation with enzymatic hydrolysis, achieving the highest extraction yield (up to 13.78%) and a shorter extraction duration compared with single-mode methods. Nevertheless, UAEE requires multi-parameter optimization, and its industrial scalability and enzyme recyclability remain major challenges.

A prominent research gap is that current studies predominantly focus on extraction yield while neglecting the impacts of different extraction technologies on the structural integrity and biological activities of PAPs. Harsh alkaline conditions or prolonged thermal treatment may alter molecular weight, monosaccharide composition, and chain conformation, thereby impairing functional properties. In addition, hybrid technologies have not been sufficiently evaluated in terms of cost-effectiveness, environmental sustainability, and compatibility with green chemistry principles. Systematic comparative investigations of thermal, non-thermal, and hybrid extraction methods under unified bioactivity evaluation systems are still lacking.

In the future, it will be possible to conduct standardized comparisons among thermal, non-thermal, and hybrid technologies using consistent structural characterization, such as molecular weight distribution and glycosidic linkage analysis, and bioactivity assays. It will be possible to develop scalable multi-field–enzyme hybrid extraction systems, such as microwave-assisted enzymatic extraction and high-pressure-assisted enzymatic extraction, for industrial translation; establish structure–activity relationships between extraction conditions and the functional properties of PAPs; and perform life-cycle assessments to optimize sustainable extraction processes.

Table 1 summarizes and compares different extraction methods for PAPs.

## 3. Purification Methods of PAPs

Crude polysaccharide extracts obtained via various methods contain impurities such as proteins, pigments, and inorganic salts, which can interfere with subsequent separation, structural analysis, and bioactivity studies [37]. Therefore, purification is an essential initial step for polysaccharide research. This crucial process entails the removal of proteins, pigments, and other low-molecular-weight impurities from complex mixtures, typically requiring a combination of techniques.

### 3.1. Deproteinization Methods of PAPs

During the extraction process of crude polysaccharides, a high protein content often interferes with subsequent research. Therefore, deproteinization becomes a necessary pretreatment step [38]. However, both proteins and polysaccharides are hydrophilic macromolecular compounds. When proteins are removed, polysaccharides are often lost at the same time, thereby affecting the yield. How to achieve efficient deproteinization and minimize polysaccharide loss has become one of the key issues in current research. At present, the commonly used deproteinization methods for PAPs mainly include the enzymatic method, the Sevag method [17], the trichloroacetic acid (TCA) method [26], etc.

Enzymatic deproteinization utilizes proteases to specifically degrade proteins, which has the advantages of high efficiency and low loss of polysaccharides. However, it has limitations such as strict reaction conditions, high enzyme costs, and easy inactivation. In contrast, the Sevag method relies on organic solvents to denature and precipitate proteins with mild conditions. It is particularly suitable for removing free proteins, but the operation is cumbersome, the efficiency is limited, and multiple treatments can easily lead to polysaccharide loss. Its effect is affected by factors such as the solution–reagent ratio, the chloroform-to-n-butanol ratio, and the oscillation time. For example, after protein removal by the Sevag method, the protein removal rate of PPS reached 86.75% [35]. The TCA method uses acidic conditions to denature and precipitate proteins. It is simple to operate and highly efficient, but the reaction is intense. If the pH is too low, it may cause acid hydrolysis of polysaccharides, which may lead to structural degradation. The above-mentioned methods each have their advantages and disadvantages. When used alone, it is often difficult to balance the efficiency of deproteinization and the retention rate of polysaccharides. Therefore, in recent years, researchers have tended to adopt combined deproteinization strategies, such as the Sevag-enzyme-linked method. Yang et al. used the Sevag-enzyme-linked method to remove proteins from PAPs, achieving a protein removal rate of 91% [18]. This approach not only reduced the protein content but also minimized the loss of polysaccharides, thereby achieving a more ideal purification effect.

### 3.2. Removing Other Magazines Methods of PAPs

When extracting polysaccharides from the flowers, stems, leaves and other parts of plants, the obtained products often contain significant amounts of pigments, which need to be decolorized to improve the purity [39]. In recent years, various decolorization methods have been widely applied in the purification process of polysaccharides, such as the macroporous resin method, activated carbon method, etc. Due to the diverse chemical structures of pigments, in practical applications, appropriate decolorization methods should be selected based on their properties. For instance, some researchers have carried out decolorization treatment on PAPs through the AB-8 macroporous resin method [32]. In addition to pigments, the crude extract of polysaccharides may also contain inorganic salts, monosaccharides, oligosaccharides and other low-molecular-weight non-polar impurities. Such small-molecule substances can usually be removed by dialysis. Dialysis bags have different molecular weight cut-off specifications. Therefore, it is necessary to select the appropriate specification of dialysis bag based on the molecular weight range of the target polysaccharide to achieve effective separation of small-molecule impurities.

### 3.3. Separation Methods of PAPs

The fractionation of polysaccharides is a crucial step for in-depth studies of their structure–activity relationships, with the aim of separating them into distinct components based on differences in molecular weight, charge, or solubility. Currently used methods are primarily based on three principles. The first and most classical method is fractional precipitation, which relies on solubility differences. This is typically achieved by the gradual addition of ethanol at varying concentrations; polysaccharides with higher molecular weights or lower solubility precipitate first. This method is straightforward and serves as a common fundamental technique in both laboratory and industrial settings. For instance, researchers have obtained two refined polysaccharides, PI and PII, through stepwise ethanol precipitation [34].

The second category includes methods based on molecular size, such as gel filtration chromatography [40]. This technique separates polysaccharides precisely by utilizing the pore size of the gel matrix, allowing larger molecules to elute first while smaller ones are retained longer, resulting in high resolution. For example, one study isolated and purified a homogeneous acidic heteropolysaccharide, PPSB, using Sepharose CL-6B gel filtration chromatography [29].

**Figure 4:**
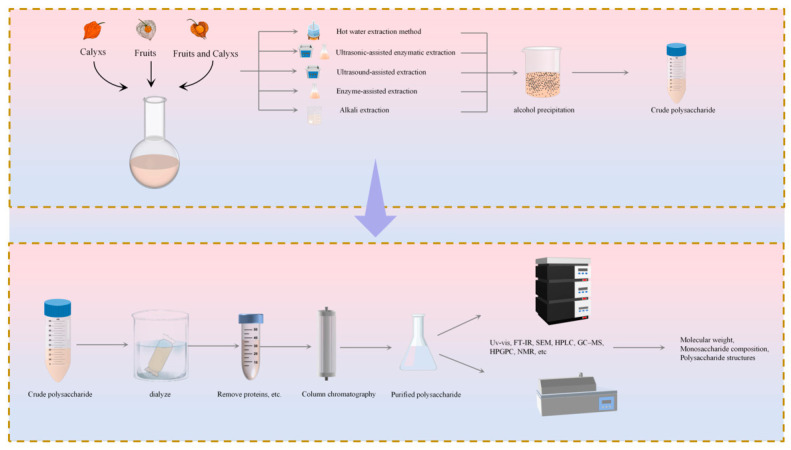
The process for extracting and purifying the PAPs.

Thirdly, fractionation based on charge properties is primarily accomplished via ion-exchange chromatography [40]. This method exploits electrostatic interactions to adsorb negatively charged acidic polysaccharides, followed by adjustment of elution conditions for separation. It is particularly suitable for the fine fractionation of polysaccharides containing uronic acids. For instance, Ge et al. obtained four PAPs using a DEAE anion-exchange chromatography column. In practical research, these methods are often employed in combination to achieve more efficient and precise separation [17]. The process for extracting and purifying the PAPs is shown in Figure 4.

### 3.4. Future Research Perspectives for PAP Purification

Current purification strategies for PAPs remain largely empirical and laboratory-oriented, with several critical bottlenecks restricting their translation toward agricultural and food science applications.

First, green, low-cost and continuous purification technologies tailored for PAPs require urgent development. Traditional organic-solvent-based deproteinization and batch-type chromatography are unsuitable for large-scale production. Future studies should focus on integrating membrane separation, magnetic-assisted purification and continuous chromatographic systems to realize high-efficiency impurity removal while maintaining native polysaccharide structure and bioactivity. Second, systematic optimization of combined purification workflows is needed to balance impurity removal efficiency, polysaccharide recovery rate and economic cost, especially targeting the co-removal of polyphenols, flavonoids and other co-extracted bioactive impurities during integrated extraction-purification processes. Third, structure-guided purification should be emphasized, linking separation parameters to polysaccharide molecular weight, monosaccharide composition and charge characteristics, to obtain homogeneous PAP fractions with defined functional properties. Fourth, more attention should be paid to food-grade purification protocols compatible with food additive standards, exploring safe, non-toxic and sustainable purification processes for developing PAP-based functional food ingredients. Collectively, these research directions will address key challenges in PAPs industrialization and promote their high-value utilization in agriculture, functional foods and related fields.

## 4. Structural Characterization of PAPs

**Figure 5:**
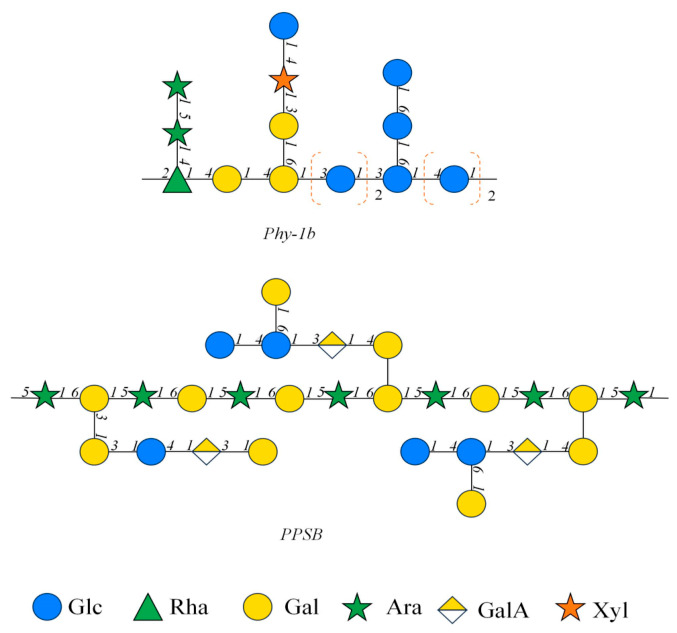
Structure of well-studied PAPs.

Polysaccharides, a class of macromolecules characterized by complex structures and diverse sources, exhibit significant variation in distribution and structure across different biological tissues [41]. Numerous studies have confirmed that polysaccharides are key active components in many medicinal plants. Their biological activity is closely related to chemical structure, which is determined by multiple parameters such as molecular weight, monosaccharide composition, glycosidic linkage types, and spatial conformation [42]. Consequently, polysaccharides extracted from different sources or plant parts often differ markedly in their structural features and functional properties. Figure 5 presents the structures of PAPs that are the primary subjects of research.

### 4.1. Molecular Weight of PAPs

Molecular weight is a key physical parameter of polysaccharides [43]. It serves not only as a fundamental criterion for distinguishing different polysaccharide components but also directly influences their solubility, viscosity, and conformational behavior in solution. Currently, common methods for determining the molecular weight of PAPs include high-performance liquid chromatography (HPLC), high-performance gel permeation chromatography (HPGPC), gel filtration chromatography (GFC), and high-performance size-exclusion chromatography (HPSEC). For example, Ge et al. extracted and purified four polysaccharide components from the calyx of *P. alkekengi*, designated as PAVF I, II-a, II-b, and III. Using GFC, they determined the molecular weights of these components to be 107 kDa, 500 kDa, 170 kDa, and 420 kDa, respectively [17]. In another study, Zhang et al. measured the molecular weights of the three polysaccharides Phy-1a, Phy-1b and Phy-1c as 59 kDa, 9.8 kDa and 9.8 kDa, respectively, by HPLC [44]. Existing research indicates that the molecular weight of PAPs is mostly distributed between 4.9 kDa and 500 kDa.

### 4.2. Monosaccharide Composition of PAPs

Monosaccharide composition is the basic unit that constitutes the primary structure of polysaccharides, and its analysis and identification are the primary steps in elucidating the structure of polysaccharides [45]. According to current research, the composition of PAPs is Rha, Ara, Gal, Glc, Xyl, Man, Rib, Fru, Sor, GalA, GlcN, GlcA, GalN·HCl, and GlcN·HCl. Generally, polysaccharide samples need to be first degraded into monosaccharide components through acid hydrolysis and then separated and identified by chromatography or electrophoresis techniques. Common methods include HPLC, gas chromatography (GC), thin-layer chromatography (TLC), and high-performance capillary electrophoresis (HPCE), etc. For instance, a study isolated and purified a uniform acidic heteropolysaccharide, PPSB. Through GC analysis, it was found that its monosaccharide composition included Ara, Gal, Glc, and GalA, with a molar ratio of 2.6:3.6:2:1 [29]. By precisely determining the category and molar ratio of monosaccharides, the connection mode of core structures such as glycosidic bonds between the main chain and side chains of polysaccharides can be effectively inferred, thereby laying a foundation for a deeper understanding of their physicochemical properties and biological activities.

### 4.3. Structural Features of PAPs

As one of the primary active components in TCM, polysaccharides possess highly complex structures that vary significantly depending on the plant parts from which they are derived [46]. This structural diversity, influenced by factors such as molecular weight, monosaccharide composition, spatial conformation, and glycosidic linkage patterns, directly determines their biological activities [47]. Therefore, conducting detailed chemical structure analyses of polysaccharides from different plant parts holds significant research value and necessity [48].

From the stems of *P. alkekengi*, researchers isolated a water-soluble polysaccharide designated as WSPA [18]. Its FT-IR spectrum displayed characteristic polysaccharide absorption bands, including a broad and strong peak between 3600 and 3200 cm^−1^ corresponding to O-H stretching vibrations, and a peak near 2930 cm^−1^ resulting from C-H stretching vibrations in the sugar rings and side chains. Notably, a distinct peak observed at 1600–1650 cm^−1^ indicated the presence of carboxyl C=O stretching vibrations characteristic of uronic acid units, which preliminarily suggested that WSPA contains uronic acid. Peaks in the 1200–1000 cm^−1^ region were associated with the stretching vibrations of C-O-C glycosidic bonds and C-O-H bonds in the sugar rings, further confirming the sample’s typical polysaccharide structure. Structural features of WSPA were elucidated through partial acid hydrolysis, periodate oxidation, Smith degradation, carboxyl reduction, and methylation analysis. The results revealed that the backbone and side chains of WSPA are composed of (1→3)-linked Glc, (1→3)-linked Gal, (1→2)-linked Xyl, (1→2)-linked Ara, and (1→2)-linked Rha. GalA was found exclusively in the main chain. All side chains of WSPA are attached to the O-2 position of (1→6)-linked Gal and are terminated by Glc at their ends.

Methylation analysis combined with GC-MS, conducted by Zhang et al. on the polysaccharide Phy-1b purified from *P. alkekengi* fruit, revealed a complex glycosidic linkage profile consisting of 12 different types. Phy-1b was mainly composed of terminal sugars (1-Ara*f*); 1,5-Ara*f*; 1,4-Xyl*p*; 1-Glc*p*; 2,4-Rha*p*; 1,3-Glc*p*; 1,4-Gal*p*; 1,4-Glc*p*; 1,3-Gal*p*; 1,6-Glc*p*; 1,3,6-Glc*p*; and 1,4,6-Gal*p*, with a molar ratio of 5.2:7.1:7.8:13.7:6.3:11.2:7.0:16.3:7.4:6.0:6.8:5.3. The complete primary structure of Phy-1b was determined by integrating multiple one- and two-dimensional NMR techniques, including ^1^H-NMR, ^13^C-NMR, COSY, HSQC, HMBC, and NOESY. Its backbone was established as →2)-*α*-L-Rha*p*-(1→4)-*β*-D-Gal*p*-(1→4)-*β*-D-Gal*p*-(1→[3)-*β*-D-Glc*p*-(1]2→3)-*β*-D-Glc*p*-(1→[4)-*β*-D-Glc*p*-(1]2→. Three side chains *β*-L-Ara*f*-(1→5)-*β*-L-Ara*f*-(1→, *β*-D-Glc*p*-(1→4)-*β*-D-Xyl*p*-(1→3)-*β*-D-Gal*p*-(1→, and *β*-D-Glc*p*-(1→6)-*β*-D-Glc*p*-(1→ are attached to the main chain via the O-4 position of →2,4)-*α*-L-Rha*p*, the O-6 position of →4,6)-*β*-D-Gal*p*, and the O-6 position of →3,6)-*β*-D-Glc*p*, respectively [44].

Ge et al. extracted and purified four polysaccharide components from the calyx of *P. alkekengi*, designated as PAVF I, II-a, II-b, and III [17]. IR analysis indicated that all components displayed characteristic polysaccharide absorption bands, including a broad peak between 3600 and 3200 cm^−1^ due to O-H stretching vibrations, a peak near 2930 cm^−1^ arising from C-H stretching vibrations, and a peak around 1600–1650 cm^−1^ that suggested the presence of C=O bonds and thus uronic acid. The region from 950 to 1200 cm^−1^ showed absorptions typical of C-O-C and C-O-H bonds, confirming their polysaccharide nature. Notably, the IR spectrum of PAVF I exhibited a distinct absorption peak at 870 cm^−1^, which was absent in the other fractions. This peak is commonly associated with either the *β*-type glycosidic linkage configuration or characteristic absorptions of mannose. This unique signal suggests that the monosaccharide composition or glycosidic bond configuration of PAVF I may be significantly different from the other three components. Its structure may contain mannose or glycosidic bonds mainly in the *β*-configuration, which may be potentially related to its strongest antioxidant activity demonstrated in subsequent studies.

The methods employed for the isolation and purification of PAPs, as well as their resultant structural characteristics such as molecular weight and monosaccharide composition, are outlined in Table 2.

## 5. Biological Activities of PAPs

**Figure 6:**
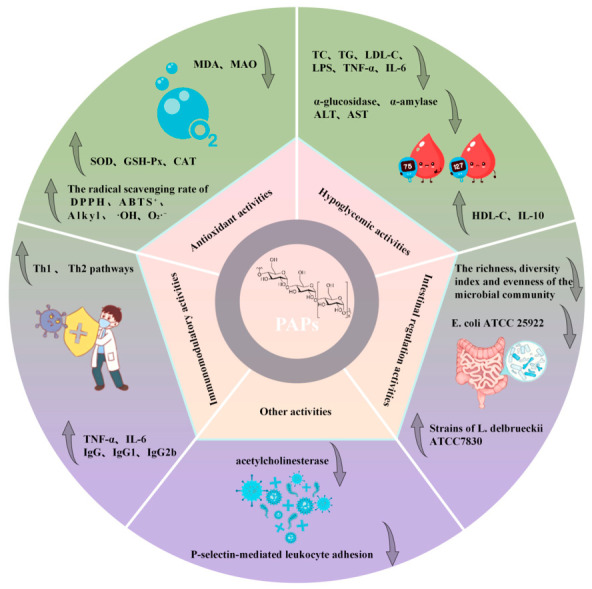
An overview of the biological activities of PAPs.

Numerous studies have demonstrated the diverse and significant biological activities of PAPs, such as antioxidant and hypoglycemic effects. An overview of these activities is presented in Figure 6.

### 5.1. Antioxidant Activities

Oxidative stress refers to a pathological state in which the generation of free radicals in the body is out of balance with the antioxidant defense system [50], leading to excessive accumulation of free radicals and subsequently causing damage to cell structure [51].

A large number of studies have shown that PAPs have good antioxidant effects. Researchers isolated four polysaccharide components, PAVFI, PAVFII-a, PAVFII-b, and PAVF III, from *P. alkekengi* and conducted antioxidant experiments on these four polysaccharide components in vitro, using Vc as the control substance. The scavenging abilities of these four polysaccharides for DPPH free radicals, hydroxyl radicals and superoxide anion radicals were determined, respectively. The results show that PAVF I has the strongest scavenging ability for DDPH, hydroxyl radicals and superoxide anion radicals, and is stronger than Vc and other components. The scavenging rates for DPPH and hydroxyl radicals reach 53.3 ± 4.1%, 80.5 ± 3.2% and 68.3 ± 2.6%, respectively. All four polysaccharides demonstrated a concentration-dependent manner, meaning that the higher the concentration of PAPs, the stronger the antioxidant capacity. Notably, PAVF I exhibited significant in vitro antioxidant activity, effectively scavenging various free radicals and outperforming Vc. This indicates its potential for development as a natural antioxidant [17].

Another study determined the antioxidant activities of *P. alkekengi* fruit polysaccharide (FP) and *P. alkekengi* fruit calyx polysaccharide (FCP) through in vitro experiments, and the anti-aging and anti-fatigue activities of FP and FCP were explored through in vivo experiments. The in vitro antioxidant experiments were conducted to determine their scavenging abilities against hydroxyl radicals, DPPH, and alkyl radicals, respectively. The results showed that their scavenging ability against hydroxyl radicals was better at low concentrations but still weaker than that of Vc. When the concentration was greater than 1 mg/mL, the scavenging ability of both FP and FCP became better than that of Vc. Furthermore, when the concentration exceeded 1.5 mg/mL, the effect of FCP was superior to that of FP, and its scavenging ability was stronger than that of Vc. Regarding the scavenging ability against DPPH, when the concentration was greater than 1.5 mg/mL, the effects of both FP and FCP were significantly better than that of Vc, and the effect of FP was consistently stronger than that of FCP. However, for the scavenging ability against alkyl radicals, both FP and FCP showed that their antioxidant capacity gradually increased with the increase of polysaccharide concentration, but their scavenging ability remained weaker than that of Vc. From these data, it can be concluded that FP and FCP, especially FP, are effective natural antioxidants, whose activity is superior to that of the common synthetic antioxidant Vc and is dose-dependent.

In vivo experiments, researchers established an aging model by injecting D-gal into mice and administering different doses of FP orally. The results showed that FP significantly enhanced the activities of SOD, GSH-Px, and CAT in aging mice and significantly reduced the levels of aging markers MDA and MAO. Subsequent in vivo anti-fatigue experiments revealed that, compared with the control group, the time for mice in each FP-administered group to reach exhaustive swimming was significantly prolonged. These findings indicate that FP is not only a potent natural antioxidant but also exhibits significant anti-aging and anti-fatigue effects in vivo, providing a scientific basis for developing PAPs into anti-aging and anti-fatigue therapeutics [33].

Tong et al. extracted and isolated four polysaccharide components from *P. alkekengi*, designated as PAPSA-a, PAPSA-b, PAPSB-c, and PAPSB-d. In vitro antioxidant experiments were performed to determine their scavenging capabilities against DPPH radicals, superoxide anion radicals, and hydroxyl radicals, respectively. The results indicated that all components exhibited concentration-dependent antioxidant activity. Among them, PAPSB-c and PAPSB-d demonstrated the strongest activity across all assays, reaching maximal scavenging capacity at a concentration of 8 mg/mL. Specifically, their DPPH scavenging rates reached 83.2% and 86.5%, with EC_50_ values of 3.6 mg/mL and 3.1 mg/mL, respectively. For superoxide anion radicals, the scavenging rates were 62.3% and 68.6%, with corresponding EC_50_ values of 4.1 mg/mL and 3.8 mg/mL. Against hydroxyl radicals, the scavenging rates were 74.1% and 77.4%, respectively. These findings demonstrate that PAPSB-c and PAPSB-d possess significant in vitro antioxidant capacity, particularly excelling in scavenging DPPH, superoxide anions, and hydroxyl radicals. Both polysaccharide components show potential for development as natural antioxidants and could be applied in functional foods or the pharmaceutical field [30].

In another study, Liu et al. isolated and purified two polysaccharide components from the stem of *P. alkekengi*, obtaining SPAP-1 and SPAP-2. In vitro experiments were conducted to determine the reducing ability of both compounds toward Fe^3+^. Simultaneously, using Vc as a reference, their scavenging abilities against DPPH radicals and ABTS^+^ radicals were evaluated. The results show that SPAP-1 has a stronger ability than SPAP-2 to reduce Fe^3+^ and scavenge both types of free radicals. Moreover, both components demonstrated concentration-dependent antioxidant activity, with their efficacy gradually increasing as concentration rose. The stronger antioxidant capacity of SPAP-1 may be attributed to its lower molecular weight and the presence of uronic acid [32].

**Figure 7:**
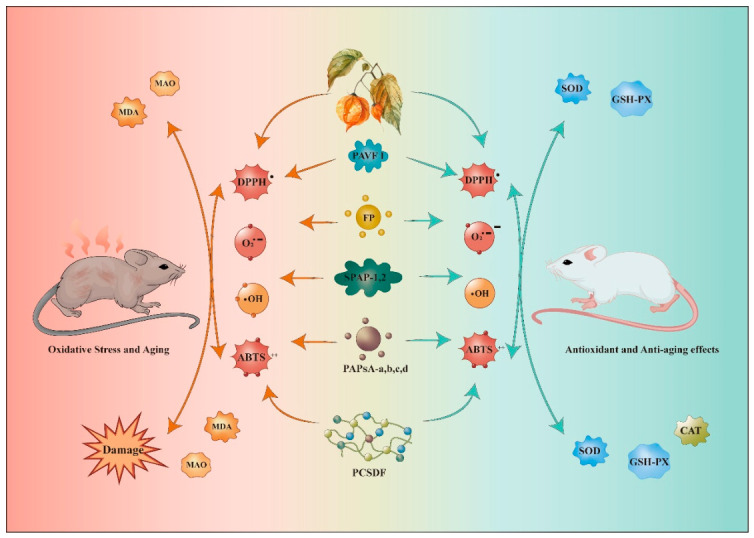
The mechanism of the antioxidant effect of PAPs.

Dietary fiber is a kind of polysaccharide [52]. It cannot be digested and absorbed by the gastrointestinal tract, nor can it produce energy [53]. Therefore, it was once regarded as a nutrient-free substance and thus received insufficient attention for a long time. However, with the in-depth development of nutrition and related sciences, people have gradually discovered that dietary fiber has considerable biological activity [54]. In a study, a dietary fiber, PCSDF, was extracted and isolated from *P. alkekengi*, and in vitro antioxidant experiments were conducted. The scavenging capabilities of PCSDF against DPPH radicals, ABTS^+^ radicals, hydroxyl radicals, and superoxide anion radicals were determined, and their effectiveness was expressed in terms of IC_50_ values. The results showed that the IC_50_ values of PCSDF for these four free radicals were 3.67, 2.96, 5.45, and 2.90 mg/mL, respectively. This study fully demonstrates that the soluble dietary fiber extracted from *P. alkekengi* possesses good antioxidant capacity and can help the body combat oxidative stress [26]. The mechanism of the antioxidant effect of PAPs is shown in Figure 7.

### 5.2. Hypoglycemic Activities

Hyperglycemia refers to the range where the glucose content in the blood is higher than the normal value [55]. In a long-term state of hyperglycemia, complications are prone to occur, seriously affecting the quality of life [56]. Researchers discovered a polysaccharide PFP from *P. alkekengi* and conducted in vivo experiments. Administration of PFP can significantly improve hyperglycemia caused by dietary AGEs in mice and alleviate lipid and insulin abnormalities. First, male BALB/c mice were divided into five groups. They were, respectively, the normal control group (NC), the model group (DA), and the treatment groups with three different doses of PFP. After ten weeks of treatment, biochemical analyses of serum and liver samples were conducted by observing the changes in body weight, food intake, fasting blood glucose levels, etc., of mice before and after administration, and the content of fecal short-chain fatty acids (SCFAs) in mouse feces was determined. The results showed that mice treated with PFP significantly reduced fasting blood glucose, decreased the contents of TC, TG and LDL-C in serum, increased HDL-C, decreased the level of serum LPS, decreased TNF-α and IL-6 in liver and serum, and increased the level of anti-inflammatory factor IL-10. From these data, we can find that PPF can improve insulin signaling and glycolipid metabolism by regulating the structure of the intestinal flora, increasing beneficial metabolites SCFAs, alleviating endotoxemia and liver inflammation, and effectively preventing insulin resistance induced by dietary AGEs. This provides a scientific basis for functional foods or adjuvant therapeutic agents for the prevention of metabolic diseases [16].

A dietary fiber, PCSDF, was extracted and isolated from *P. alkekengi*. Beyond its established antioxidant activity, subsequent in vitro studies have further assessed the potential of PCSDF in lowering blood glucose and blood lipids. For the hypoglycemic evaluation, its inhibitory activities against α-glucosidase and α-amylase were determined. For the hypolipidemic assessment, its inhibitory activity against pancreatic lipase was measured. The results demonstrated that PCSDF exhibited inhibitory effects with IC_50_ values of 1.20 mg/mL and 9.21 mg/mL against α-glucosidase and α-amylase, respectively, and an IC_50_ value of 16.95 mg/mL against pancreatic lipase. These data indicate that PCSDF possesses notable potential for lowering both blood glucose and blood lipids [26].

**Figure 8:**
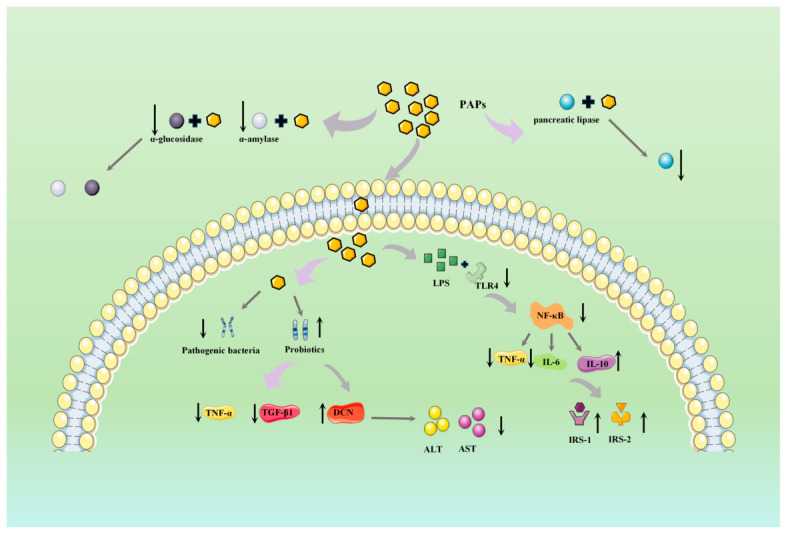
The mechanism of the hypoglycemic activities of PAPs.

Type 2 diabetes mellitus (T2DM) is a chronic disease primarily caused by insufficient insulin secretion or reduced insulin utilization [57], and it has become one of the three major chronic diseases threatening human health [58]. In one study, a polysaccharide named PPSB was isolated from *P. alkekengi* and was found to exhibit a good therapeutic effect on type 2 diabetes, as well as to alleviate diabetes-induced liver damage. Specifically, KM mice were first induced with streptozotocin to establish a type 2 diabetes model. They were then divided into five groups: the blank control group, the diabetes model group, the positive control group, the low-dose PPSB group, and the high-dose PPSB group. All groups received continuous intragastric administration for 5 weeks. The results showed that PPSB could significantly reduce blood glucose levels in diabetic mice. In the high-dose group, blood glucose decreased from 25.38 ± 2.21 mmol/L to 18.01 ± 2.53 mmol/L, with a significant effect observed from the third week onward. Meanwhile, PPSB also significantly reduced serum levels of ALT and AST, indicating a protective effect against liver injury [59]. The regulation mechanism of PAPs on blood glucose is shown in Figure 8.

### 5.3. Immunomodulatory Activities

Immunomodulatory function is an essential mechanism for maintaining bodily health, and dysfunction of the immune system can readily lead to various diseases [60]. Numerous studies have found that PAPs possess notable immunomodulatory effects. For instance, Yang et al. extracted and isolated a homogeneous polysaccharide, PPSB, from the fruit of *P. alkekengi* and investigated its immunostimulatory activity on RAW264.7 macrophages. RAW264.7 cells were treated with different concentrations of PPSB, namely 40, 80, and 160 µg/mL for 24 h, respectively, and treated with 1 µg/mL LPS as positive controls. Cell proliferation was assessed using the MTT assay. Subsequent experiments, including measurements of reactive oxygen species (ROS), nitric oxide (NO), TNF-*α*, and interleukin-6 (IL-6) production, revealed that PPSB at 80 and 160 µg/mL significantly promoted the proliferation of RAW264.7 cells. Furthermore, PPSB dose-dependently enhanced the secretion of TNF-*α* and IL-6. These results demonstrate that PPSB can comprehensively activate RAW264.7 macrophages by enhancing their proliferation, phagocytic capacity, and production of key immune mediators. This study provides a molecular pharmacological basis for the potential use of PAPs as innate immunomodulators [61].

**Figure 9:**
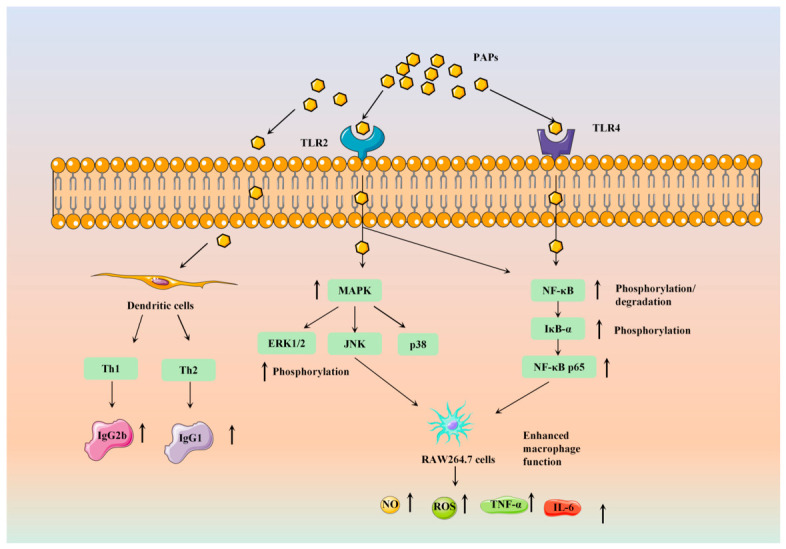
The mechanism of the immunomodulatory activities of PAPs.

Researchers studied the isolation and purification of a polysaccharide called WSPA from *P. alkekengi* and evaluated its immune-enhancing effect as an adjuvant for DNA vaccines [18]. First, the ICR mice were grouped. Each mouse was intramuscularly injected with 10 µg of pD-HSP90C for modeling. Meanwhile, different doses of WSPA, 5, 10, and 20 µg per mouse, were administered for treatment as the experimental group. PBS and pcDNA3.1 were used as controls. After immunization, in vivo electroporation technology was used to enhance DNA uptake. The immune serum was collected and the specific antibody levels against epitope C (LKVIRK) were detected by ELISA and Western blot methods. Western blot results showed that serum from mice in the pD-HSP90C group and the pD-HSP90C combined with WSPA group could specifically recognize the recombinant protein rP-HSP90C, proving that the vaccine successfully stimulated specific antibodies. The control group showed no such reactivity. Further quantitative analysis by ELISA indicated that pD-HSP90C itself stimulated the production of specific IgG, IgG1, and IgG2b antibodies. After combination with WSPA, especially at doses of 10 µg and 20 µg, the titers of all detected antibody types were significantly increased. These findings demonstrate that WSPA can synergistically enhance the immunogenicity of the DNA vaccine. WSPA can serve as an effective DNA vaccine adjuvant, significantly enhancing the vaccine-specific antibody response while simultaneously promoting both Th1 and Th2 immune pathways. This provides an important candidate substance and a theoretical basis for developing new vaccine adjuvants based on plant polysaccharides. The mechanism of the immunomodulatory activities of PAPs is shown in Figure 9.

### 5.4. Intestinal Regulation Activities

In existing studies, PPSB has been found to exhibit a good hypoglycemic effect. Additionally, research indicates that PPSB also has the function of regulating intestinal flora. By collecting feces from the five groups of mice in the previous experiments, total genomic DNA was extracted from the fecal samples using a fecal DNA kit. DNA fragments of the same length but with different sequences were separated using urea and formamide at different concentrations, thereby reflecting the diversity of the microbiota. The results showed that compared with the normal group, the richness, diversity index, and evenness of the microbiota in the diabetes model group were significantly reduced. The PPSB treatment groups, especially the high-dose group, alleviated this imbalance to a certain extent, although the microbiota did not fully return to the normal level. Therefore, PPSB can significantly reshape the intestinal microbiota structure of type 2 diabetic mice, promoting recovery toward a healthier state, supporting the growth of beneficial bacteria, and inhibiting the proliferation of potentially harmful bacteria [61].

Li et al. investigated the regulatory effect of PPSB on intestinal flora through both in vivo and in vitro experiments [62]. In the in vitro experiment, strains of Lactobacillus debrueckii ATCC 7830 and *Escherichia coli* ATCC 25922 were selected to examine the influence of different concentrations of PPSB on their growth curves. The results revealed that only at concentrations between 12.5 and 25.0 mg/mL did PPSB promote the growth of Lactobacillus delbrueckii ATCC 7830, while it exerted a concentration-dependent inhibitory effect on *Escherichia coli* ATCC 25922 at all tested concentrations. For the in vivo experiment, an antibiotic-induced intestinal flora imbalance model was established by intragastric administration of levofloxacin to female BALB/c mice. The mice were randomly divided into a normal control group, a levofloxacin model group, and several PPSB treatment groups receiving different doses. Fecal samples were collected, and total bacterial DNA was extracted. The V3 hypervariable region of the bacterial 16S rRNA gene was amplified by PCR using primers GC 357f and 518r. The amplified products were subsequently analyzed by DGGE. Specific bands from the DGGE gel were excised, recovered, reamplified, and sequenced. The obtained sequences were compared by BLAST against the NCBI database to identify the key bacterial genera (blast.ncbi.nlm.nih.gov/Blast.cgi (accessed on 4 June 2026)). Finally, based on the DGGE banding profiles, the Shannon–Wiener diversity index and evenness index E were used to quantitatively assess the diversity and uniformity of the microbial community. DGGE profiles and cluster analysis indicated that levofloxacin significantly altered the structure of the intestinal flora in mice. In contrast, the flora structure in the PPSB-treated groups, especially the high-dose group, resembled that of the normal group more closely. Since levofloxacin led to an abnormal increase in flora diversity, PPSB could partially restore the balance. PAPs can effectively ameliorate antibiotic-induced intestinal microecological imbalance, which provides an important scientific basis for developing PAPs-based natural functional foods or drugs aimed at preventing or treating antibiotic-associated diarrhea and microbial dysbiosis.

### 5.5. Other Activities

A large number of studies have demonstrated that PAPs exhibit a range of biological activities, including antioxidant and immunomodulatory activities. Beyond these, PAPs possess additional biological properties. For instance, SPAP-1 and SPAP-2 have shown potent antioxidant activity. Furthermore, based on the Ellman assay, researchers evaluated their inhibitory effects on acetylcholinesterase (AChE). The results indicated that the IC_50_ values of SPAP-1 and SPAP-2 for AChE inhibition were 31.350 μM and 23.768 μM, respectively. Both polysaccharides exhibited concentration-dependent inhibition of AChE, with the highest inhibition rates of 38.41% and 32.92% achieved at a concentration of 3 mg/mL. These findings suggest that SPAP-1 and SPAP-2 possess the potential to enhance cholinergic system function and cognitive ability, offering novel insights for their potential application in combating Alzheimer’s disease [31].

Inflammation is a defensive response of the body to stimuli, clinically manifested as redness, swelling, heat, pain, and impaired function [63]. Studies have found that PAPs possess good anti-inflammatory effects. Researchers isolated and purified three polysaccharide components from the roots of *P. alkekengi*, namely PPS-1, PPS-2, and PPS-3, and evaluated their inhibitory effects on P-selectin-mediated leukocyte adhesion [30].

Flow cytometric assays revealed that PPS-2 had the strongest dose-dependent inhibitory effect on the binding of P-selectin to surface ligands on HL-60 cells. At a concentration of 5 mg/mL, its inhibition rate was as high as 85.2%. In contrast, PPS-1 only exhibited moderate inhibitory activity with an inhibition rate of 50.9%, while the inhibitory effect of PPS-3 was weak with an inhibition rate of 24.5%. In the adhesion assay under static conditions, PPS-2 again demonstrated significant inhibitory ability, with inhibition rates of 54.1% and 71.3% at concentrations of 1 mg/mL and 5 mg/mL, respectively. PPS-1 showed a considerable inhibitory effect only at a concentration of 5 mg/mL, with an inhibition rate of 56.3%, whereas PPS-3 did not show a significant inhibitory effect at all tested concentrations. Further, in laminar flow assays, PPS-2 still demonstrated outstanding inhibitory efficacy, reducing the rolling and adhesion rate of HL-60 cells by 79.1% at 5 mg/mL, indicating that it can effectively block the function of P-selectin even in a dynamic fluid environment. In comparison, the inhibitory effect of PPS-1 was significantly weakened, with an inhibition rate of less than 30%, while PPS-3 was almost inactive.

In summary, PPS-2 demonstrated potent and concentration-dependent P-selectin antagonistic effects in all three experiments, maintaining high activity especially under physiologically relevant flow conditions. This suggests that it is a promising candidate molecule from natural sources for anti-inflammatory development. The summary of the biological activities of PAPs is shown in Table 3.

## 6. Structure–Activity Relationship of PAPs

The biological activity of polysaccharides largely depends on their structural characteristics. Parameters such as monosaccharide composition [64], molecular weight [65], glycosidic bond type [66], and spatial conformation collectively regulate their bioactive effects. In some studies, four polysaccharide components were isolated, designated as PAVF I, II-a, II-b, and III, with molecular weights of 107 kDa, 500 kDa, 170 kDa, and 420 kDa, respectively [17]. Monosaccharide composition analysis revealed that PAVF I consists mainly of arabinose; PAVF II-a primarily contains xylose, glucose, and fructose; PAVF II-b is rich in rhamnose, mannose, and fructose; while PAVF III is a proteoglycan whose principal monosaccharides include rhamnose, fructose, glucose, and galactose. Results from antioxidant activity assays showed that PAVF I exhibited the strongest antioxidant activity across all tested concentrations, even surpassing that of the positive control, vitamin C. This finding indicates that the antioxidant capacity of polysaccharides is closely related to their molecular weight, monosaccharide composition, and structural features. Components with lower molecular weight and a high arabinose content may interact more readily with free radicals, thereby demonstrating stronger activity. Furthermore, the presence of uronic acid may further enhance the antioxidant mechanism by improving solubility and altering charge properties.

In another study, two polysaccharide components, SPAP-1 and SPAP-2, were isolated and purified from the stem of *P. alkekengi*, and their antioxidant capacities were evaluated [32]. The molecular weights of SPAP-1 and SPAP-2 were 9.1 kDa and 13.5 kDa, respectively. In assays assessing DPPH and ABTS free radical scavenging activity as well as iron-reducing power, SPAP-1 demonstrated stronger antioxidant capacity. This observation is consistent with most studies, namely that polysaccharides with lower molecular weights tend to exhibit better solubility and more exposed active sites, thereby facilitating electron or hydrogen atom transfer reactions with free radicals. Furthermore, galacturonic acid was detected in SPAP-1, whereas no uronic acid was found in SPAP-2. The presence of uronic acid introduces carboxyl groups, which can impart a negative charge to the polysaccharide and enhance its metal ion chelating ability. This, in turn, improves its reducing power and free radical scavenging efficiency.

At present, research on the structure–activity relationship of PAPs mainly focuses on the influence of basic structural parameters such as monosaccharide composition, molecular weight, and glycosidic bond type on their biological activity. These works provide an important basis for understanding the functional characteristics of polysaccharides. However, the regulatory mechanisms of its biological activity by the related spatial conformations remain insufficiently studied and have not yet formed a systematic understanding. Therefore, future research should strengthen the application of solution conformational analysis techniques such as X-ray small-angle scattering, nuclear magnetic resonance and cryoelectron microscopy, combined with activity detection and conformational parameter correlation analysis, to systematically clarify the intrinsic connection between the advanced structural features of brocade PAPs and their biological activities. This not only deepens the understanding of the structure–activity relationship of polysaccharides, but also provides a structural basis for the modification of active polysaccharides, promoting their application and development in functional foods and drug adjuvants.

## 7. Application of PAPs in Foods

*P. alkekengi* is not only utilized in TCM but also bears edible fruits with a sweet and sour flavor. The fruits are rich in vitamin C, vitamin E, *β*-carotene, and various trace elements [67]. Owing to its rich bioactive components, *P. alkekengi* shows broad application prospects in food, pharmaceutical, and resource utilization sectors [68]. For instance, its fruit can be processed into effervescent tablets via an acid–base mixed granulation process. Under optimal conditions, the resulting product disintegrates rapidly and possesses a unique flavor. It also holds potential for the development of functional beverages and similar products, offering a new direction for natural and healthy foods [69].

PAPs exhibit a variety of biological activities, such as antioxidant, immunoenhancing, and intestinal flora-regulating effects. These properties position PAPs as promising natural preservatives, antioxidants, or functional food additives. The application performance of PAPs varies greatly in different food matrices due to differences in physicochemical environments, which requires targeted research for practical industrial utilization [70]. In liquid food matrices including fruit beverages and plant-based drinks, PAPs can effectively delay oxidation browning and improve product stability, which has been verified in the development of Physalis-based compound beverages [71]. In high-protein dairy matrices such as fermented yogurt, PAPs can enhance nutritional value and sensory quality by regulating intestinal flora, while matrix pH and protein–polysaccharide interactions affect their functional stability [72]. In baked foods subjected to high-temperature processing, thermal conditions may reduce the antioxidant activity of PAPs, thus limiting their direct application without protective treatment [73]. Studies have demonstrated that PAPs can serve as an effective adjuvant for nucleic acid vaccines to enhance immune responses [18]. This unique physiological activity underpins their intrinsic value and indicates significant potential for transformation and application in the future nutraceutical and functional food industry [74].

The above findings indicate that PAPs have considerable application potential in diverse food matrices, but their practical industrial translation is still restricted by matrix-specific challenges. Future research should focus on several key directions to promote the practical application of PAPs in the food industry. First, systematic studies should be conducted to clarify the influence of different food matrix environments on the structure and functional activity of PAPs and establish a theoretical basis for targeted application. Second, it is necessary to develop efficient protective technologies, such as microencapsulation and composite modification, to improve the stability of PAPs in harsh processing conditions, especially in high-temperature baked foods and acidic beverage matrices. Third, more research should be carried out on the industrial-scale application of PAPs, optimizing addition dosage, processing parameters, and application processes to balance functional effects, product quality, and economic benefits. Fourth, further exploration of the synergistic effects between PAPs and other natural bioactive components in food matrices can help develop high-value functional food products with multiple health benefits. Finally, attention should be paid to the safety evaluation and regulatory standards of PAPs as food additives, ensuring their safe and standardized application in the food industry. With the deepening of these research directions, PAPs are expected to be widely applied in various food products, realizing their high-value utilization and providing new ideas for the development of natural, healthy, and functional foods.

## 8. Toxicology of PAPs

In recent years, growing public health awareness and expanding research on edible and medicinal plants have drawn significant attention to the development and application of their active ingredients [75]. To ensure the safety and reliability of these natural products, systematic toxicological evaluation is therefore essential.

The biosafety of the polysaccharide PPSB, isolated from *P. alkekengi*, was assessed via hemolysis and acute toxicity testing [62]. In the hemolysis assay, the positive control adjuvant QuilA showed an HD of 17.11 ± 0.30 μg/mL, while PPSB displayed no hemolytic activity even at a concentration of 1000 μg/mL. In the acute toxicity study, ICR mice received subcutaneous injections of PPSB at doses up to 400 mg/kg and were monitored for 14 days. No adverse effects were observed, including mortality, swelling at the injection site, or hair loss. Histopathological examination further revealed no abnormal lesions in the kidneys, livers, or spleens of PPSB-treated mice, with tissue morphology consistent with that of the saline control group. Together, these findings demonstrate that PPSB exhibits a favorable safety profile.

Existing research mainly focuses on the extraction, purification and various biological activities of PAPs. However, this is in sharp contrast to the scarcity of basic toxicological data. Therefore, to ensure safety, it is necessary to conduct a systematic toxicological evaluation of PAPs. In the future, chronic toxicity assays on PAPs should be conducted to verify the safety of repeated administration, thereby ensuring the safety of subsequent clinical translation and applications.

## 9. Conclusions and Prospects

As a commonly used TCM, *P. alkekengi* contains polysaccharide components that represent important bioactive substances with broad pharmacological effects and development potential. This paper reviews the extraction, separation, purification, and pharmacological activities of PAPs. Current research indicates that water extraction and alcohol precipitation remain the primary methods for obtaining these polysaccharides. However, the application of modern techniques such as ultrasonic-assisted and enzyme-assisted extraction has significantly improved extraction yields. In separation and purification, methods including DEAE-52 cellulose ion-exchange column chromatography and Sephadex G-100 purification have successfully isolated multiple polysaccharide fractions, such as S-PMCP-1 to S-PMCP-4, from the calyx of *P. alkekengi*. Pharmacological studies have confirmed that these polysaccharides exhibit various biological activities, including antioxidant and immunomodulatory effects, demonstrating their promising application prospects in medicine and healthcare.

Despite significant advances in PAPs research, a number of challenges persist. First, the structure–activity relationship remains insufficiently clarified. Although preliminary insights into the primary structure have been obtained, research on higher-order structures and their associations with pharmacological activity remains limited. In particular, the specific active sites and mechanisms of different polysaccharide components have yet to be fully elucidated. This lack of clarity hinders the targeted development and clinical translation of PAPs. Second, the depth of pharmacological research is still limited. For instance, although PAPs are known to exert anti-inflammatory effects by inhibiting the NF-*κ*B signaling pathway, the precise molecular targets and pathway networks require further exploration. Studies on in vivo metabolism, bioavailability, and potential toxicity are also relatively scarce. Third, current preparation technologies face issues of high cost and low efficiency, making it difficult to meet industrial-scale demands. The transition from laboratory-scale to industrial production requires further process optimization. Additionally, resource utilization remains suboptimal, as insufficient research has been conducted on the distribution and activity of polysaccharides in different plant parts such as the fruit, calyx, and rhizome.

Accordingly, future research should focus on the following directions. One key area is to deepen investigations into the structure–activity relationships. Advanced techniques such as chromatographic separation, nuclear magnetic resonance, mass spectrometry, and X-ray diffraction could be comprehensively applied to systematically analyze the fine structure of PAPs, including glycosidic linkage patterns, branching degrees, and three-dimensional conformation. Clarifying the structural basis of their pharmacological activity through structure–activity relationship studies would provide a theoretical foundation for the targeted design and optimization of PAP-based drugs. It is worth noting that studies suggest the anti-inflammatory activity of *P. alkekengi* calyx polysaccharides may be related to their triple-helix structure, which merits further investigation. Another important direction is expanding research on pharmacological mechanisms. Beyond existing work on antioxidant and immunomodulatory effects, the potential activities of PAPs in anti-tumor and neuroprotective applications should be further explored. Multiomics approaches such as genomics and proteomics could help reveal its mechanisms at a systems level. Concurrently, greater emphasis should be placed on in vivo efficacy evaluation and pharmacokinetic studies to provide a stronger scientific basis for preclinical development. Given the traditional use of *P. alkekengi* in treating inflammatory conditions such as sore throat and pharyngitis, in-depth research into its anti-inflammatory mechanisms holds significant theoretical and practical value.

## Figures and Tables

**Table 1:** Comparison of different extraction methods for PAPs.

Method	Source	Extraction Condition	Total Yield	Evaluation of the Method	Ref.
HWE	Fruit calyx	6 h, 90 °C, 1:15 g/mL	2.92%	The operation is simple, the process is mature, the cost is low, the solvent is safe and readily available, environmentally friendly, and free from organic solvent pollution. However, it is inefficient, time-consuming, has poor selectivity and may damage the structure.	[17]
	Fruit	3 h, 100 °C, 3 times	10.96%		[16]
	Fruit	3 h, 100 °C, 3 times	0.71%		[29]
	Root	3 h, 80 °C, 3 times	8.7%		[30]
	Fruit	3 h, 80 °C, 3 times	0.7%		[31]
	Stem	1 h, 90 °C, 3 times, 1:40 *w*/*v*	N/A		[32]
	Fruit	2 h, 60–70 °C, 3 times	9.3 g/kg		[33]
	Fruit calyx	2 h, 60–70 °C, 3 times	28.3 g/kg		[33]
	Stem	2 h, 100 °C, 1:10 g/mL, 2 times	6.8%		[34]
	Fruit	3.3 h, 82 °C, 1:29 g/mL	7.92%		[35]
	Fruit calyx	2 h, 80 °C, 1:20 g/mL	N/A		[36]
UAE	Fruit calyx	25 min, 1:15 g/mL, ultrasonic power 300 W	3.67%	The extraction efficiency is extremely high, significantly shortening the time. The extraction temperature is low, which is conducive to protecting heat-sensitive components and has a high yield. However, it may produce free radicals, generate noise and is not suitable for industrial production.	[17]
EAE	Fruit calyx	1 h, 60 °C, papain 1%, pH 6.5	8.581%	The conditions are mild, the selectivity is high, the efficiency is high, and the yield is high. However, the cost of enzymes is high, the reaction time is long, and enzymes may remain in the final product.	[17]
UAEE	Fruit calyx	α-amylase 2000 U/mL, dispase 20,000 U/mL, amyloglucosidase 40,000 U/mL, ultrasonic power 300 W, 1:30 g/mL	8.74%	It has a significant synergistic effect, extremely high efficiency, increased extraction rate, mild reaction conditions, reduced enzyme dosage and energy consumption. However, the process is complex, optimization is difficult, and the cost is high, making it unsuitable for industrial production.	[26]
	Fruit	30 min, ultrasound at 50 °C, 1:15.6 g/mL, cellulase 3.4%, and 60 min, enzymatic hydrolysis at pH 5.0	13.78%		[25]
AEM	Stem	3 h, 100 °C, 0.1 mol/L NaOH, 3 times	3.4%	It has a high yield and low cost, and can be used to extract specific types of polysaccharides in a targeted manner. However, the conditions are harsh, which may seriously damage the glycosidic bonds, change the original structure of the polysaccharides, cause pollution, and be corrosive to the equipment.	[18]

N/A: information was not available.

**Table 2:** Isolation, purification and structural characterization of PAPs.

No.	Compound Name	Separation and Purification		Mw (kDa)	Chemical Structures		Monosaccharide Composition	Ref.
		Remove Impurities	Purification		Method	Glycan Characteristics		
1	PAVF I	Sevag method, dialyze	DEAE-52, Sephadex G-200	107	UV, IR, GC	N/A	Rha:Ara:Man:Glc:Gal = 12.01:60.36:2.95:9.93:9.78	[17]
2	PAVF II-a	Sevag method, dialyze	DEAE-52, Sephadex G-200	500	UV, IR, GC	N/A	Xyl:Glc:Fru = 41.61:20.73:37.2	[17]
3	PAVF II-b	Sevag method, dialyze	DEAE-52, Sephadex G-200	170	UV, IR, GC	N/A	Rha:Man:Glc:Fru:Sor = 38.77:19.3:7.39:27.01:6.18	[17]
4	PAVF III	Sevag method, dialyze	DEAE-52, Sephadex G-200	420	UV, IR, GC	N/A	Rha:Glc:Fru:Gal = 46.45:18.07:21.02:13.26	[17]
5	Phy-1a	dialyze	DEAE Sepharose Fast Flow, BRT-GS gel purification system	59	N/A	N/A	N/A	[44]
6	Phy-1b	dialyze	DEAE Sepharose Fast Flow, BRT-GS gel purification system	9.8	HPLC, GC/MS, NMR	The main chain is →2)-*α*-L-Rha-(1→4)-*β*-D-Gal-(1→4)-*β*-D-Gal-(1→[3)-*β*-D-Glc-(1]2→3)-*β*-D-Glc-(1→[4)-*β*-D-Glc-(1]2→, the branched chains are *β*-L-Ara-(1→5)-*β*-L-Ara-(1→, *β*-D-Glc-(1→4)-*β*-D-Xyl-(1→3)-*β*-D-Gal-(1→, and *β*-D-Glc-(1→6)-*β*-D-Glc-(1→	Rha:Ara:Gal:Glc:Xyl = 3.0:19.8:47.5:20.9:8.8	[44]
7	Phy-1c	dialyze	DEAE Sepharose Fast Flow, BRT-GS gel purification system	9.8	HPLC, GC-MS	N/A	Rha:Ara:Gal:Glc:Xyl:Man:Rib:GalN·HCl:GlcN·HCl = 10.4:7.9:22.8:30.5:4.6:4.4:19.4:3.9:5.8	[44]
8	PPSB	Combined enzymatic-Sevag method, dialyze	Sepharose CL-6B	27	GC, GC-MS, IR, HPLC	(1→5)-linked Ara, (1→6)-linked Gal, three branches attached to O-3 of (1→6)-linked Gal	Ara:Gal:Glc:GalA = 2.6:3.6:2:1	[29]
9	PPS-1	Combined enzymatic-Sevag method, dialyze	DEAE, Sephacryl S-200	83.1	GC, FT-IR, GC-MS, HPGPC, UV	(1→3)-linked Ara, (1→4)-linked Man, (1→3)-linked Gal, (1→4)-linked and (1→4,6)-linked Glc	Fru:Ara:Xyl:Man:Gal:Glc:GalA = 0.7:1.1:0.2:0.6:1:4.3:0.2	[30]
10	PPS-2	Combined enzymatic-Sevag method, dialyze	DEAE, Sephacryl S-200	24.7	GC, FT-IR, GC-MS, HPGPC, UV	(1→4,6)-linked Man, (1→3,6)-linked Gal, (1→4)-linked and (1→4,6)-linked Glc	Fru:Xyl:Man:Gal:Glc:GalA = 1.4:0.4:1.1:1:3.1:0.5	[30]
11	PPS-3	Combined enzymatic-Sevag method, dialyze	DEAE, Sephacryl S-200	7.5	GC, FT-IR, GC-MS, HPGPC, UV	(1→3)-linked Ara, (1→4)-linked Xyl, (1→3)-linked Gal, and (1→4)-linked and (1→4,6)-linked Glc	Fru:Ara:Xyl:Gal:Glc = 0.2:1.7:1.6:1:3.6	[30]
12	PI	Sevag method	Sephadex G-200	330.156	HPLC, IR, NMR	N/A	Man:GlcA:GalA:Glc:Gal:Xyl:Ara = 1.00:14.94:7.22:48.45:12.62:6.96:3.23	[34]
13	PII	Sevag method	Sephadex G-200	172.9	HPLC, IR, NMR	N/A	Man:Rha:GlcA:Glc:Gal:Ara = 1.00:2.58:2.22:7.04:3.11:1.35	[34]
14	S-PMCP-1	Sevag method, dialyze	DEAE-52, Sephadex G-100	16	UV-VIS, FT-IR, HPLC, NMR	N/A	Sor:Glc:GalA:GlcN:Man:Rib = 15.78:13.16:9.75:5.27:5.15:1	[36]
15	S-PMCP-2	Sevag method, dialyze	DEAE-52, Sephadex G-100	71	UV-VIS, FT-IR, HPLC, NMR	N/A	GlcN:Sor:Glc:Rib:GalA:Rha = 9.96:5.19:4.31:1.28:1.27:1	[36]
16	S-PMCP-3	Sevag method, dialyze	DEAE-52, Sephadex G-100	77	UV-VIS, FT-IR, HPLC, NMR	N/A	Glc:GalA:GlcN:Rib:GlcA:Sor:Rha:Man = 6.76:3.74:3.67:3.24:3.13:2.57:1.07:1	[36]
17	S-PMCP-4	Sevag method, dialyze	DEAE-52, Sephadex G-100	299	UV-VIS, FT-IR, HPLC, NMR	N/A	GlcA:Glc:GalA:Rib:Man:Rha:Sor = 6.99:2.79:2.67:2.13:1.28:1.05:1	[36]
18	PAPSA-a	Combined enzymatic-Sevag method, dialyze	DEAE–cellulose, Sephacryl S-300	30.6	HPSEC, HPLC, GC	N/A	Rha:Ara:Xyl:Man:Gal:Clc:GalA = 0.6:1.2:0.2:0.5:1:4.1:0.1	[31]
19	PAPSA-b	Combined enzymatic-Sevag method, dialyze	DEAE-cellulose, Sephacryl S-300	13.4	HPSEC, HPLC, GC	N/A	Rha:Ara:Man:Gal:Clc = 0.3:0.5:0.2:1:3.3	[31]
20	PAPSA-c	Combined enzymatic-Sevag method, dialyze	DEAE-cellulose, Sephacryl S-300	21.5	HPSEC, HPLC, GC	N/A	Rha:Man:Gal:Clc:GalA = 0.8:1.1:1:2.2:2.4	[31]
21	PAPSA-d	Combined enzymatic-Sevag method, dialyze	DEAE–cellulose, Sephacryl S-300	9.1	HPSEC, HPLC, GC	N/A	Rha:Man:Gal:Glc:GalA = 0.5:0.4:1:2.7:1.9	[31]
22	SPAP-1	AB-8 macroporous resin, Sevag method	Sephadex G-200, DEAE-52	9.1	UV-vis, HPGPC, FT-IR, NMR, SEM	the microstructure of SPAP-1 appeared loose and irregular with frond-like and chain-branch style,	Rha:Ara:GalA:Man:Glc = 1.0:1.5:1.9:29.1:1.03	[32]
23	SPAP-2	AB-8 macroporous resin, Sevag method	Sephadex G-200, DEAE-52	13.5	UV-vis, HPGPC, FT-IR, NMR, SEM	SPAP-2 exhibited a dense sheet-like and blocky appearance with a smooth surface.	Rha:Xyl:Ara:Man:Glc = 31.8:14.8:1.0:6.5:22.6	[32]
24	PCSDF	TCA method	N/A	N/A	UV-vis, FT-IR, SEM, HPLC	the structure of PCSDF is composed of several round spherical structures, and the surface is relatively rough	Man:GlcA:Rha:GalA:Glc:Xyl:Gal:Ara:Fru = 17.72:1.93:9.92:2.71:31.07:19.65:1.90:14.81:0.30	[26]
25	PPS	X-5 macroporous resin, Sevag method	N/A	N/A	HPLC	N/A	Man:Rha:GlcA:GalA:Glc:Gal:Xyl:Ara = 1.29:1.50:0.24:0.18:6.61:0.47:1.70:1.13	[35]
26	PAF-I(a)	D101 macroporous resin, Combined enzymatic-Sevag method, dialyze	N/A	4.919	HPLC, HPGPC, UV, IR	N/A	Man:Rha:GalA:Glc:Gal:Ara = 7.7:1.3:32.4:5.1:2.3:5.2	[49]
27	PAF-II(a)	D101 macroporous resin, Combined enzymatic-Sevag method, dialyze	N/A	109.441	HPLC, HPGPC, UV, IR	N/A	Man:Rha:GalA:Glc:Gal:Xyl = 1.12:0.25:0.54:1.22:0.54:1.02	[49]
28	PAF-III(a)	D101 macroporous resin, Combined enzymatic-Sevag method, dialyze	N/A	124.527	HPLC, HPGPC, UV, IR	N/A	Rha:GlcA:GalA:Glc:Gal = 7.1:4.2:5.5:14.2:5.9	[49]
29	WSPA	Combined enzymatic-Sevag method, dialyze	QAE Sephadex A-25	31	GC, HPLC, GC-MS	(1→3)-linked Glc, (1→3)-linked Gal, (1→2)-linked Xyl, (1→2)-linked Ara, (1→2)-linked Rha, terminated with Glc	Rha:Ara:Xyl:Gal:Glc:GalA = 1.0:2.5:0.8:2.7:4.4:1.4	[18]

N/A: information was not available.

**Table 3:** Summary of the biological activities of PAPs.

Biological Activities	Polysaccharide Name	Study Design	Models	Results	Ref.
Antioxidant activities	PAVF I, PAVF II-a, PAVF II-b, PAVF III	In vitro	DPPH, ·O_2_^−^, ·OH	The activities of PAVF I, II-a, and II-b are all higher than those of Vc. Among them, PAVF I has the best effect, while PAVF III shows relatively lower scavenging activity and is weaker than Vc.	[17]
	PAPSA-a, PAPSA-b, PAPSB-c, PAPSB-d	In vitro	DPPH, ·O_2_^−^, ·OH	The scavenging effects of PAPSB-c and PAPSB-d were significantly better than those of PAPSA-a and PAPSA-b.	[31]
	FP, FCP	In vitro	DPPH, ·OH, Alkyl radical	The clearance effects of FP and FCP on DPPH and ·OH are concentration-dependent. The clearance activity of FP on DPPH is stronger than that of FCP.	[33]
	FP, FCP	In vivo	D-galactose-induced aging mouse	↑The activities of SOD, GSH-Px, and CAT, ↓MDA, MAO.	[33]
	PCSDF	In vitro	DPPH, ABTS+, ·OH, ·O_2_^−^	The IC_50_ values for the scavenging rates against DPPH, ABTS^+^, ·OH, and ·O_2_^−^ were 3.67, 3.67, 5.45, and 2.90 mg/mL, respectively.	[26]
Hypoglycemic activities	PFP	In vivo	Dietary AGE-induced mice	↓HOMA-IR, ↑HOMA-IS, ↓LPS, TLR4, TNF-α, IL-6, ↑IL-10.	[16]
	PPSB	In vivo	Streptozotocin-induced diabetic mice	↓Blood sugar level, expression of TGF-β1 and TNF-α.	[59]
	PCSDF	In vitro	In vitro inhibition models	The IC_50_ values for α-glucosidase inhibition, α-amylase inhibition, and pancreatic lipase inhibition were 1.20, 9.21, and 16.95 mg/mL, respectively.	[26]
Immunomodulatory activities	PPSB	In vitro	RAW264.7 cells	↑The proliferation of RAW264.7 cells, ↑NO, ROS, TNF-α, IL-6.	[61]
	WSPA	In vivo	DNA vaccine-induced ICR mice	↑Titers of specific antibodies IgG, IgG1 (Th2-type), and IgG2b (Th1-type)	[18]
Intestinal regulation activities	PPSB	In vivo	Streptozotocin-induced diabetic mice	↑Probiotics	[59]
	PPSB	In vitro, In vivo	Lactobacillus debrueckii ATCC 7830, *Escherichia coli* ATCC 25922, levofloxacin-induced mouse	In vitro, PPSB promoted the growth of Lactobacillus at 12.5–25.0 mg/mL; in vivo, PPSB partially restored antibiotic-induced gut microbiota dysbiosis.	[62]
Other activities	PPS-1, PPS-2, PPS-3	In vitro	HL-60 cells, CHO-P cells	PPS-2 is the most effective of the three fractions in blocking P-selectin-mediated leukocyte adhesion, showing potent anti-inflammatory potential.	[30]
	SPAP-1, SPAP-2	In vitro	acetylcholinesterase inhibitory model	Both exhibited concentration-dependent activity, with SPAP-1 being superior to SPAP-2. At 3.0 mg/mL, the inhibition rates were 38.41% and 32.92%, respectively.	[31]

↑: improve or promote. ↓: inhibit or reduce.

## Data Availability

No new data were created or analyzed in this study. Data sharing is not applicable to this article.

## Abbreviations

The following abbreviations are used in alphabetical order:

AChE	Acetylcholinesterase
AEM	Alkali extraction method
Ara	Arabinose
EAE	Enzymatic extraction
FP	*P. alkekengi* fruit polysaccharide
FCP	*P. alkekengi* fruit calyx polysaccharide
Fru	Fructose
Gal	Galactose
GalA	Galacturonic acid
GalN·HCl	Galactosamine hydrochloride
GC	Gas chromatography
GFC	Gel filtration chromatography
Glc	Glucose
GlcA	Glucuronic acid
GlcN	D-glucosamine
GlcN·HCl	Glucosamine hydrochloride
HPLC	High-performance liquid chromatography
HWE	Hot water extraction
HPGPC	High-performance gel permeation chromatography
HPSEC	High-performance size-exclusion chromatography
IL-6	Interleukin-6
Man	Mannose
NO	Nitric oxide
NMR	Nuclear magnetic resonance
PAPs	Polysaccharides from *P. alkekengi*
RSM	Response surface methodology
Rha	Rhamnose
TCA	Trichloroacetic acid
TCM	Traditional Chinese medicine
TLC	Thin-layer chromatography
T2DM	Type 2 diabetes mellitus
UAE	Ultrasonic-assisted extraction
UAEE	Ultrasonic-assisted enzymatic extraction
ROS	Reactive oxygen species
Rib	Ribose
Sor	Sorbitol
Xly	Xylose

## References

[1] Yari P., Alirezalu A., Khalili S. (2025). A comparative study of chemical composition, phenolic compound profile and antioxidant activity of wild grown, field and greenhouse cultivated *Physalis* (*P. alkekengi* and *P. peruviana*). Food Prod. Process. Nutr..

[2] Teng Y., Zhang X.R., Li X.L., Gao J., Ni L., Zhan G.Q. (2024). Two new sesquiterpenoid glycoside compounds from the calyces of *Physalis alkekengi* L. var. *franchetii* (Mast.) Makino. J. Asian Nat. Prod. Res..

[3] Helvacı S., Kökdil G., Kawai M., Duran N., Duran G., Güvenç A. (2010). Antimicrobial activity of the extracts and physalin D from *Physalis alkekengi* and evaluation of antioxidant potential of physalin D. Pharm. Biol..

[4] Zhang W.N., Tong W.Y. (2016). Chemical Constituents and Biological Activities of Plants from the Genus *Physalis*. Chem. Biodivers..

[5] Liang L., Li C., Wang Y., Yue Y., Zhang H., Yang M., Cao X., Zhao M., Du J., Peng M. (2022). *Physalis alkekengi* L. var. *franchetii* (Mast.) Makino: A review of the pharmacognosy, chemical constituents, pharmacological effects, quality control, and applications. Phytomedicine.

[6] Wen X., Erşan S., Li M., Wang K., Steingass C.B., Schweiggert R.M., Ni Y., Carle R. (2019). Physicochemical characteristics and phytochemical profiles of yellow and red *Physalis* (*Physalis alkekengi* L. and *P. pubescens* L.) fruits cultivated in China. Food Res. Int..

[7] Zhao J., Zhang X.R., Wu Y., Liu Y.L., Liang Y.F., Teng Y. (2025). Three New Physalins from *Physalis alkekengi* L. var. *franchetii* (Mast.) Makino. Molecules.

[8] Liu Y., Wang X., Li C., Yu D., Tian B., Li W., Sun Z. (2023). Research progress on the chemical components and pharmacological effects of *Physalis alkekengi* L. var. *franchetii* (Mast.) Makino. Heliyon.

[9] Park H.J., Shim H.S., Han A.R., Seo E.K., Kim K.R., Han B.H., Shim I. (2022). Anti-Inflammatory Effect of Three Isolated Compounds of *Physalis alkekengi* var. *franchetii* (PAF) in Lipopolysaccharide-Activated RAW 264.7 Cells. Curr. Issues Mol. Biol..

[10] Popova V., Petkova Z., Mazova N., Ivanova T., Petkova N., Stoyanova M., Stoyanova A., Ercisli S., Okcu Z., Skrovankova S. (2022). Chemical Composition Assessment of Structural Parts (Seeds, Peel, Pulp) of *Physalis alkekengi* L. Fruits. Molecules.

[11] Li A.L., Chen B.J., Li G.H., Zhou M.X., Li Y.R., Ren D.M., Lou H.X., Wang X.N., Shen T. (2018). *Physalis alkekengi* L. var. *franchetii* (Mast.) Makino: An ethnomedical, phytochemical and pharmacological review. J. Ethnopharmacol..

[12] Shu Z., Xing N., Wang Q., Li X., Xu B., Li Z., Kuang H. (2016). Antibacterial and Anti-Inflammatory Activities of *Physalis alkekengi* var. *franchetii* and Its Main Constituents. Evid.-Based Complement. Altern. Med..

[13] Zhang C.Y., Luo J.G., Liu R.H., Lin R., Yang M.H., Kong L.Y. (2016). ^1^H NMR spectroscopy-guided isolation of new sucrose esters from *Physalis alkekengi* var. *franchetii* and their antibacterial activity. Fitoterapia.

[14] Cao M., Yang S., Tao A., Li J. (2025). Advancements in the extraction, characterization and function activities of polysaccharides from *Physalis* L.: A review. Int. J. Biol. Macromol..

[15] Wang Y., Zhen Y., Xing M. (2021). Research on the Production Process of *Physalis alkekengi* L. var. *franchetii* (Mast.) Makino. Agric. Prod. Process..

[16] Wu Y., Dong L., Song Y., Wu Y., Zhang Y., Wang S. (2022). Preventive effects of polysaccharides from *Physalis alkekengi* L. on dietary advanced glycation end product-induced insulin resistance in mice associated with the modulation of gut microbiota. Int. J. Biol. Macromol..

[17] Ge Y., Duan Y., Fang G., Zhang Y., Wang S. (2009). Polysaccharides from fruit calyx of *Physalis alkekengi* var. francheti: Isolation, purification, structural features and antioxidant activities. Carbohydr. Polym..

[18] Yang J., Yang F., Yang H., Wang G. (2015). Water-soluble polysaccharide isolated with alkali from the stem of *Physalis alkekengi* L.: Structural characterization and immunologic enhancement in DNA vaccine. Carbohydr. Polym..

[19] Ai X., Yu P., Hou Y., Shu B., Han B., Yang M., Fan X., Wang J. (2025). Research progress in the extraction, purification, structural features, biological activities, and structure-activity relationships from Prunella vulgaris polysaccharides. Int. J. Biol. Macromol..

[20] Guo Y., Qin W., Hou Y., Zhu W., Zhao H., Zhang X., Jiao K. (2025). Extraction, purification, structural characteristics and biological properties of the polysaccharides from Rubus L: A review. Food Chem..

[21] Xue H., Gao Y., Wu L., Cai X., Liao J., Tan J. (2025). Research progress in extraction, purification, structure of fruit and vegetable polysaccharides and their interaction with anthocyanins/starch. Crit. Rev. Food Sci. Nutr..

[22] Xu Y., Cao H., He J. (2025). Research advances in okra polysaccharides: Green extraction technology, structural features, bioactivity, processing properties and application in foods. Food Res. Int..

[23] Liu Y., Huang G. (2019). Extraction and derivatisation of active polysaccharides. J. Enzyme Inhib. Med. Chem..

[24] Wen L., Zhang Z., Sun D.W., Sivagnanam S.P., Tiwari B.K. (2020). Combination of emerging technologies for the extraction of bioactive compounds. Crit. Rev. Food Sci. Nutr..

[25] Yan P., Zhang Y., Shen X., Zhang M. (2017). Ultrasonic-assisted enzymatic hydrolysis of Calyx Seu Fructus *Physalis* for extraction of fructification polysaccharide. Sci. Technol. Chem. Ind..

[26] Wang J., Liang B., Li Z., Wu X., Wang Z., Yu T., Gao Y., Dai Y., Wu Q. (2024). Extraction, characterization, and bioactivity of soluble dietary fiber from *Physalis alkekengi* L. calyx. J. Food Meas. Charact..

[27] Khedmat L., Izadi A., Mofid V., Mojtahedi S.Y. (2020). Recent advances in extracting pectin by single and combined ultrasound techniques: A review of techno-functional and bioactive health-promoting aspects. Carbohydr. Polym..

[28] He Z., Zhu Y., Bao X., Zhang L., Li N., Jiang G., Peng Q. (2019). Optimization of Alkali Extraction and Properties of Polysaccharides from *Ziziphus jujuba cv.* Residue. Molecules.

[29] Tong H., Liang Z., Wang G. (2008). Structural characterization and hypoglycemic activity of a polysaccharide isolated from the fruit of *Physalis alkekengi* L. Carbohydr. Polym..

[30] Tong H., Wang R., Liu X., Wang G., Du F., Zeng X. (2011). Structural characterization and in vitro inhibitory activities in P-selectin-mediated leukocyte adhesion of polysaccharide fractions isolated from the roots of *Physalis* alkekengi. Int. J. Biol. Macromol..

[31] Tong H., Zhu M., Feng K., Sun L. (2011). Purification, characterization and in vitro antioxidant activities of polysaccharide fractions isolated from the fruits of *Physalis alkekengi* L. J. Food Biochem..

[32] Liu X., Bian J., Li D., Liu C., Xu S., Zhang G., Zhang L., Gao P. (2019). Structural features, antioxidant and acetylcholinesterase inhibitory activities of polysaccharides from stem of *Physalis alkekengi* L. Ind. Crops Prod..

[33] Ge Y., Duan Y., Fang G., Zhang Y., Wang S. (2009). Study on biological activities of *Physalis alkekengi* var. francheti polysaccharide. J. Sci. Food Agric..

[34] Zhou Z., Jiao L., Yu M., Dai W., Zhao C. (2012). Studies on Preparation and Structure of Homogeneous Polysaccharides P_I_, P_II_ from Rhizome of *Physalis alkekengi* var. franchetii. J. Jilin Agric. Univ..

[35] Yuan Q. (2024). Research on the Component Analysis and Effects on Cognitive Dysfunction in Mice of the Polysaccharides from the Fruits of *Physalis alkekengi* L. var. *franchetii* (Mast.) Makino. Master’s Thesis.

[36] Tao S. (2024). Study on the Isolation, Purification, Structural Characterization and Anti-Inflammatory Effects of Polysaccharides from Fruit Calyx of *Physalis alkekengi* L. var. *franchetii* (Mast.) Makino. Master’s Thesis.

[37] Simayi Z., Rozi P., Yang X., Ababaikeri G., Maimaitituoheti W., Bao X., Ma S., Askar G., Yadikar N. (2021). Isolation, structural characterization, biological activity, and application of *Glycyrrhiza* polysaccharides: Systematic review. Int. J. Biol. Macromol..

[38] Wang M., Li C., Li J., Hu W., Yu A., Tang H., Li J., Kuang H., Zhang H. (2023). Extraction, Purification, Structural Characteristics, Biological Activity and Application of Polysaccharides from *Portulaca oleracea* L. (Purslane): A Review. Molecules.

[39] Sun M., Zhang Y., Gao W., He Y., Wang Y., Sun Y., Kuang H. (2024). Polysaccharides from *Porphyra haitanensis*: A Review of Their Extraction, Modification, Structures, and Bioactivities. Molecules.

[40] Xue H., Wang W., Bian J., Gao Y., Hao Z., Tan J. (2022). Recent advances in medicinal and edible homologous polysaccharides: Extraction, purification, structure, modification, and biological activities. Int. J. Biol. Macromol..

[41] Seidi F., Yazdi M.K., Jouyandeh M., Habibzadeh S., Munir M.T., Vahabi H., Bagheri B., Rabiee N., Zarrintaj P., Saeb M.R. (2022). Crystalline polysaccharides: A review. Carbohydr. Polym..

[42] Rußler A., Bogolitsyna A., Zuckerstätter G., Potthast A., Rosenau T. (2012). Chemical characterization of polysaccharides. The European Polysaccharide Network of Excellence (EPNOE) Research Initiatives and Results.

[43] Koh H.S.A., Lu J., Zhou W. (2019). Structure characterization and antioxidant activity of fucoidan isolated from *Undaria pinnatifida* grown in New Zealand. Carbohydr. Polym..

[44] Zhang Y., Wen X., Xu N., Fu H., Lv G., Yu W., Wei L., Zhao L. (2025). Structural Identification of *Physalis alkekengi* L. Polysaccharides. Molecules.

[45] Yao L., Zhu L., Chen C., Wang X., Zhang A., Gao S., Wu J., Qin L. (2024). A systematic review on polysaccharides from fermented Cordyceps sinensis: Advances in the preparation, structural characterization, bioactivities, structure-activity relationships. Int. J. Biol. Macromol..

[46] Zeng P., Li J., Chen Y., Zhang L. (2019). The structures and biological functions of polysaccharides from traditional Chinese herbs. Prog. Mol. Biol. Transl. Sci..

[47] Shi L., He Q., Li J., Liu Y., Cao Y., Liu Y., Sun C., Pan Y., Li X., Zhao X. (2024). Polysaccharides in fruits: Biological activities, structures, and structure-activity relationships and influencing factors—A review. Food Chem..

[48] Muhidinov Z.K., Nasriddinov A.S., Strahan G.D., Jonmurodov A.S., Bobokalonov J.T., Ashurov A.I., Zumratov A.H., Chau H.K., Hotchkiss A.T., Liu L.S. (2024). Structural analyses of apricot pectin polysaccharides. Int. J. Biol. Macromol..

[49] Gao J. (2024). Isolation, Purification and Hypoglycaemic Activity of Polysaccharides from the Calyx of *Physalis alkekengi* L. var. *franchetii* (Mast.) Makino. Master’s Thesis.

[50] Pizzino G., Irrera N., Cucinotta M., Pallio G., Mannino F., Arcoraci V., Squadrito F., Altavilla D., Bitto A. (2017). Oxidative Stress: Harms and Benefits for Human Health. Oxid. Med. Cell. Longev..

[51] Sies H. (2015). Oxidative stress: A concept in redox biology and medicine. Redox Biol..

[52] Tian S., Chu Q., Ma S., Ma H., Song H. (2023). Dietary Fiber and Its Potential Role in Obesity: A Focus on Modulating the Gut Microbiota. J. Agric. Food Chem..

[53] Guan Z.W., Yu E.Z., Feng Q. (2021). Soluble Dietary Fiber, One of the Most Important Nutrients for the Gut Microbiota. Molecules.

[54] Ranganathan N., Anteyi E. (2022). The Role of Dietary Fiber and Gut Microbiome Modulation in Progression of Chronic Kidney Disease. Toxins.

[55] Tabák A.G., Herder C., Rathmann W., Brunner E.J., Kivimäki M. (2012). Prediabetes: A high-risk state for diabetes development. Lancet.

[56] Hammer M., Storey S., Hershey D.S., Brady V.J., Davis E., Mandolfo N., Bryant A.L., Olausson J. (2019). Hyperglycemia and Cancer: A State-of-the-Science Review. Oncol. Nurs. Forum.

[57] Galicia-Garcia U., Benito-Vicente A., Jebari S., Larrea-Sebal A., Siddiqi H., Uribe K.B., Ostolaza H., Martín C. (2020). Pathophysiology of Type 2 Diabetes Mellitus. Int. J. Mol. Sci..

[58] Sampath Kumar A., Maiya A.G., Shastry B.A., Vaishali K., Ravishankar N., Hazari A., Gundmi S., Jadhav R. (2019). Exercise and insulin resistance in type 2 diabetes mellitus: A systematic review and meta-analysis. Ann. Phys. Rehabil. Med..

[59] Zhao X., Chen Z., Yin Y., Li X. (2017). Effects of polysaccharide from *Physalis alkekengi* var. francheti on liver injury and intestinal microflora in type-2 diabetic mice. Pharm. Biol..

[60] Li L., Kou Z., Zhao F., Wang Y., Zhang X. (2024). Network meta-analysis of four common immunomodulatory therapies for the treatment of patients with thin endometrium. Gynecol. Endocrinol..

[61] Yang F., Li X., Yang Y., Ayivi-Tosuh S.M., Wang F., Li H., Wang G. (2019). A polysaccharide isolated from the fruits of *Physalis alkekengi* L. induces RAW264.7 macrophages activation via TLR2 and TLR4-mediated MAPK and NF-κB signaling pathways. Int. J. Biol. Macromol..

[62] Li Y., Han S., Zhao H., Liu Y., Fang J., Wang G. (2011). Evaluation of immunologic enhancement mediated by a polysaccharide isolated from the fruit of *Physalis alkekengi* L. var. *francheti* (Mast.) *Makino*. J. Med. Plants Res..

[63] Kirsch-Volders M., Bolognesi C., Ceppi M., Bruzzone M., Fenech M. (2020). Micronuclei, inflammation and auto-immune disease. Mutat. Res.-Rev. Mutat. Res..

[64] Wang Z., Zheng Y., Lai Z., Hu X., Wang L., Wang X., Li Z., Gao M., Yang Y., Wang Q. (2024). Effect of monosaccharide composition and proportion on the bioactivity of polysaccharides: A review. Int. J. Biol. Macromol..

[65] Xia C., Xu X., Zhang R., Su D., Jia X., Deng M., Lee Y.K., Zhang M., Huang F. (2025). Effects of molecular weight on simulated digestion and fecal fermentation of polysaccharides from longan pulp in vitro. Int. J. Biol. Macromol..

[66] Yuan Q., Liu W., Hao W., Chen Y., Xiao Y., Li H., Shui M., Wu D.T., Wang S. (2025). Glycosidic linkages of fungus polysaccharides influence the anti-inflammatory activity in mice. J. Adv. Res..

[67] Xiang Y., Zang H., Xiang Y., Yin L. (2018). Processing Technology of Canned Fructus *Physalis*. J. Yulin Coll..

[68] Mirzaee F., Saeed Hosseini A., Askian R. (2019). Therapeutic activities and phytochemistry of *Physalis* species based on traditional and modern medicine. Res. J. Pharmacogn..

[69] Ma H., Zhao L., Dong H., Cai Q. (2024). Study on the Preparation Process of *Physalis alkekengi* Effervescent Tablets. J. Liaoning Univ. TCM.

[70] Shenstone E., Lippman Z., Van Eck J. (2020). A review of nutritional properties and health benefits of *Physalis* species. Plant Foods Hum. Nutr..

[71] Wang S., Guan Q., Zhang T., Yang C. (2022). Development of Compound Functional Beverage of *Physalis alkekengi* and *Aronia melanocarpa*. Storage Process.

[72] Zhang J., Xu N., Yu W., Lv G., Song Y., Yang J. (2024). Fuzzy mathematics combined with response surface method to optimize theprocess of polysaccharide from *Physalis alkekengi* L yogurt. China Dairy Ind..

[73] Wen X., Hempel J., Schweiggert R.M., Ni Y., Carle R. (2017). Carotenoids and Carotenoid Esters of Red and Yellow *Physalis* (*Physalis alkekengi* L. and *P. pubescens* L.) Fruits and Calyces. J. Agric. Food Chem..

[74] Dellafiora L., Dall’Asta C. (2017). Forthcoming Challenges in Mycotoxins Toxicology Research for Safer Food-A Need for Multi-Omics Approach. Toxins.

[75] Schoental R. (1965). Toxicology of natural products. Food Cosmet. Toxicol..

